# Associations Between Traumatic Stress, Brain Volumes and Post-traumatic Stress Disorder Symptoms in Children: Data from the ABCD Study

**DOI:** 10.1007/s10519-021-10092-6

**Published:** 2021-12-03

**Authors:** Daniel Bustamante, Ananda B. Amstadter, Joshua N. Pritikin, Timothy R. Brick, Michael C. Neale

**Affiliations:** 1 Virginia Institute for Psychiatric and Behavioral Genetics, 800 E Leigh Street, Biotech One, Box 980126, Richmond, VA 23298 USA; 2 Integrative Life Sciences Doctoral Program, Virginia Commonwealth University, Richmond, VA USA; 3 Department of Psychiatry, School of Medicine, Virginia Commonwealth University, Richmond, VA USA; 4 Department of Human Development and Family Studies, and Institute for Computational and Data Sciences, The Pennsylvania State University, University Park, PA USA

**Keywords:** Genetic, Environment, Brain, Regularization, PTSD, Children, MRI

## Abstract

Reduced volumes in brain regions of interest (ROIs), primarily from adult samples, are associated with posttraumatic stress disorder (PTSD). We extended this work to children using data from the Adolescent Brain Cognitive Development (ABCD) Study® (N = 11,848; M_age_ = 9.92). Structural equation modeling and an elastic-net (EN) machine-learning approach were used to identify potential effects of traumatic events (TEs) on PTSD symptoms (PTSDsx) directly, and indirectly via the volumes 300 subcortical and cortical ROIs. We then estimated the genetic and environmental variation in the phenotypes. TEs were directly associated with PTSDsx (r = 0.92) in children, but their indirect effects (r < 0.0004)—via the volumes of EN-identified subcortical and cortical ROIs—were negligible at this age. Additive genetic factors explained a modest proportion of the variance in TEs (23.4%) and PTSDsx (21.3%), and accounted for most of the variance of EN-identified volumes of four of the five subcortical (52.4–61.8%) three of the nine cortical ROIs (46.4–53.3%) and cerebral white matter in the left hemisphere (57.4%). Environmental factors explained most of the variance in TEs (C = 61.6%, E = 15.1%), PTSDsx (residual-C = 18.4%, residual-E = 21.8%), right lateral ventricle (C = 15.2%, E = 43.1%) and six of the nine EN-identified cortical ROIs (C = 4.0–13.6%, E = 56.7–74.8%). There is negligible evidence that the volumes of brain ROIs are associated with the indirect effects of TEs on PTSDsx at this age. Overall, environmental factors accounted for more of the variation in TEs and PTSDsx. Whereas additive genetic factors accounted for most of the variability in the volumes of a minority of cortical and in most of subcortical ROIs.

**Supplementary Information:**

The online version contains supplementary material available at 10.1007/s10519-021-10092-6.

## Introduction

Exposure to traumatic events (TEs) is common, with approximately 80% of the US population having experienced at least one during their lifetime (Breslau 2009). TEs are also common during childhood (Farrugia et al. 2011) and are necessary but not sufficient risk factors for developing posttraumatic stress disorder (PTSD) and symptoms (PTSDsx) thereof (Cloitre et al. 2009). Subtypes of TEs include physical maltreatment or abuse, accidents, and witnessing violence with a prevalence of 9% to 70% depending on the subtype (Saunders and Adams 2014). However, meeting PTSD diagnosis after experiencing TEs is less common (Breslau 2009; Perkonigg et al. 2000; Santiago et al. 2013), and the risk varies depending on the type of trauma. Greater risk for developing PTSD is linked to: (i) exposure to TEs during childhood or adolescence as opposed to exclusively during adulthood (Kulkarni et al. 2013); and (ii) interpersonal trauma (IPT; e.g., sexual and physical assault; Forbes et al. 2012; Thege et al. 2017; Weaver and Clum 1995) compared to experiencing accidental TEs (e.g., natural disasters, motor vehicle accident) or witnessing them. Beyond type of trauma experienced, neurobiological (Akiki et al. 2017; Cross et al. 2017; Herringa 2017) and genetic factors (Nievergelt et al. 2019; Nugent et al. 2008; Sheerin et al. 2017) are also associated with risk of PTSD.

Volumes of subcortical regions of interest (ROIs) such as the hippocampus, amygdala and caudate nucleus (Hanson et al. 2015; Herringa et al. 2012; Logue et al. 2018), and of cortical ROIs such as the prefrontal cortex, caudal anterior cingulate, insula, and ventromedial prefrontal cortex (Chao et al. 2013; De Bellis et al. 2002; Morey et al. 2016) have been associated with increased risk for PTSD. However, studies distinguishing the direction of causation between experienced TEs and variation of volume in ROIs, and between the latter and PTSDsx, have found varied results and have mostly focused on the hippocampus (Gilbertson et al. 2002; Karl et al. 2006; Smith 2005). Genetic and neurobiological risk factors underly the complex etiology of PTSD. Investigative approaches that model both unidirectional and reciprocal influences among its components can help to deepen the understanding of this complex etiology, and at early stages of life (e.g., childhood). Few studies have jointly collected data on structural magnetic resonance imaging (MRI; sMRI) and TEs or PTSD (Aupperle et al. 2012; De Bellis et al. 2001; Heyn et al. 2019; Papagni et al. 2011). Studies have largely used a hypothesis-driven approach in selecting ROIs for analysis. Moreover, most have examined adults’ retrospective reports of their childhood trauma (Admon et al. 2013; Chao et al. 2014; Shin et al. 2006), with a few neuroimaging studies assessing children (Herringa 2017; Keding and Herringa 2015; Lupien et al. 2011). The present study extends the literature by proposing an agnostic analytical framework for assessing: (i) the direct effects of TEs on PTSDsx; and (ii) the indirect effects of TEs on PTSDsx via the volumes of brain ROIs. These analyses are applied to the Adolescent Brain Cognitive Development (ABCD) Study® data from children at baseline, and have potential for future investigations as data for new time points are released.

Both genetic and environmental factors are associated with the etiology and development of PTSD. The phenotypic variance can be partitioned in additive genetic, shared- and unique-environmental factors under the classical twin design, modeling data from monozygotic and dizygotic twin pairs (Neale and Cardon 1992). Twin studies have estimated that TEs and PTSD are moderately heritable (53% and 38–49% respectively; Amstadter et al. 2012; Stein et al. 2002; Wolf et al. 2018), with approximately 33% of the variance in TEs and 51% in PTSD accounted for by unique-environmental factors (Wolf et al. 2018). Furthermore, several studies have estimated that genetic factors influence 0.28–0.90 of the total variance of subcortical brain volumes (Christova et al. 2021; Schmitt et al. 2007) and substructures (Patel et al. 2017; Whelan et al. 2016). Cortical volume heritability estimates (2–75%) tend to be lower than those of subcortical volumes (Christova et al. 2021; Patel et al. 2018). More studies examining the heritability of volume in brain ROIs and PTSD development, and the contributions to these phenotypes from environmental factors during childhood and adolescence are needed. Efforts from large consortia (e.g., The Enhancing Neuro Imaging Genetics through Meta-Analysis, ENIGMA Consortium; Thompson et al. 2020) are approaching the subject. However, while using twin and single nucleotide polymorphisms (SNPs) data, the focus of consortia studies remain on adult samples (Brainstorm Consortium 2018; Hibar et al. 2015; Lee et al. 2016; Thompson et al. 2020; Whelan et al. 2016), and on brain volumes and PTSD separately with a few exceptions (e.g., Logue et al. 2018; van der Merwe et al. 2019).

The first aim of this study is to assess the direct effects of TEs on PTSDsx, and indirectly through the volumes of 300 brain ROIs (see Supplementary Information 1a and 1b for the variables’ names under subcortical and cortical datasets, respectively) in children. Hypothesis 1 is that, in children 9–11 years of age, the number of TEs directly increases the likelihood for developing a greater number of PTSDsx. Second, Hypothesis 2, is that TEs indirectly affect PTSDsx via the volume of subcortical and cortical ROIs. The second aim is to estimate the genetic and environmental influences on these phenotypes. Hypothesis 3 is that additive genetic factors account for more variation in the volumes of these brain ROIs than in TEs and PTSDsx. A corollary of Hypothesis 3 is that shared- and unique-environmental factors explain a higher proportion of the phenotypic variability in TEs and PTSDsx than of the ROIs volumes. Although not specifically an aim, variance components estimates of TEs, PTSDsx and EN-selected ROIs obtained from the full sample fit will be contrasted with those from the twin sample fit to potentially aid with interpretation of results.

## Method

### Sample, recruitment, and assessments

#### Sample

Participants (*N* = 11,848, *mean-age* = 9.92 [+ / − 0.62]) and assessments came from the ABCD Study®. The ABCD Study® is a longitudinal assessment of brain development and health of children in the US, consisting of 21 sites across the four major regions in the country (Northeast, South, Midwest and West; Dick et al. 2021; Iacono et al. 2018; Volkow et al. 2018). The sample is diverse in terms of sex (*F* = 47.86%, *M* = 52.14%) and race/ethnicity (*White* = 52.07%, *African-American* = 15.00%, *Hispanic or Latino* = 20.30%, *Asian* = 2.13%, and *All-Other* = 10.50%). Sex at birth and race/ethnicity were reported by the parent via the ABCD demographic survey. The distributions of these variables match the sociodemographic variation in the US present in the American Community Survey. The full sample includes unrelated participants (*N* = 8753, *mean-age* = 9.88 [+ / − 0.61], *F* = 47.22%, *M* = 52.78%, *White* = 49.37%, *African-American* = 15.22%, *Hispanic or Latino* = 22.46%, *Asian* = 2.53%, and *All-Other* = 10.43%), siblings (*N* = 1585, *mean-age* = 9.87 [+ / − 0.73], *F* = 49.53%, *M* = 50.47%, *White* = 51.45%, *African-American* = 15.09%, *Hispanic or Latino* = 19.76%, *Asian* = 2.15%, and *All-Other* = 11.56%) and same-sex monozygotic (MZ) and dizygotic (DZ) twin pairs. Zygosity was determined by identity-by-descent using pi-hat values (*MZ* = 0.89–1.00, *DZ* = 0.40–0.60). The analyses based on the twin sample (*twinN* = 1510, *N-MZ* = 660, *N-DZ* = 850, *mean-age* = 10.13 [+ / − 0.55], *F* = 49.48%, *M* = 50.52%, *White* = 66.12%, *African-American* = 13.89%, *Hispanic or Latino* = 10.34%, *Asian* = 0.17%, and *All-Other* = 9.47%) include complete same-sex twin pairs from the four ABCD Study® twin-hub sites (University of Colorado Boulder; University of Minnesota, Minneapolis; Virginia Commonwealth University, Richmond; Washington University, St. Louis, Missouri) only. The framework proposed in this study uses the ABCD baseline data, and is part of a larger project of our group that will implement longitudinal modeling as the subsequent ABCD data releases become available.

#### Recruitment

Probability sampling, mailing lists, referrals, and summer recruitment were used to contacting eligible families. Children and their parents were contacted via the schools proximal to each of the 21 ABCD Study® sites. Recruitment of twin pairs was conducted using twin birth registries from States of each twin-hub site, partially differing from the recruitment of singletons at each site (for more details, see Iacono et al. 2018).

#### Assessments

The baseline data release includes over 60,000 variables of mental health, substance use, neurocognition, brain imaging, genomic and environmental measures. Lifetime history of TEs (the exposure variable) and current PTSDsx (the outcome variable, based on the previous 12 months; Barch et al. 2018) from children 9 to 11 years of age, were measured using the Kiddie Schedule for Affective Disorders and Schizophrenia Parent Diagnostic Interview for The Diagnostic and Statistical Manual of Mental Disorders, Fifth Edition (DSM-5; KSADS-5; American Psychiatric Association 2013). The KSADS-5 had good to excellent Kappa coefficients (Cohen 1960), and the concurrence of diagnostic categories between the computer self-administered and the paper–pencil clinician-administered versions ranged from 88 to 96% (Barch et al. 2018). The TEs variable was based on 17 binary items comprising the DSM-5 PTSD criterion A. The PTSDsx variable included these items under criterion A, in addition to 22 binary items corresponding to the other DSM-5 PTSD diagnosis criteria. Exclusion criteria: PTSD diagnosis presupposes the exposure to at least one TE as a necessary criterion. Therefore, participants who did not endorse at least one TE (7359 at baseline; 61.95%) were coded as missing in the PTSDsx variable without omitting these participants, whose TEs and volumes of brain ROIs data were retained in the analysis.

#### Structural MRI volume variables

These variables were 42 and 258 continuous measures of volume of ROIs in mm^3^ from the subcortical and cortical sets, respectively (Hagler Jr et al. 2019). Variables of volumes of cortical ROIs were based on Desikan (Desikan et al. 2006), Destrieux (Destrieux et al. 2010) and Fuzzy-clusters (Chen et al. 2012) parcellations ROIs. The three cortical parcellations include ROI segmentations of the cortex, and including the three parcellations in analyses may seem unnecessary at first glance. However, including all the available variables from these parcellations for selection via a machine learning approach such as regularization (see *Regularization* section) can ensure a comprehensive and diverse perspective considering the complex cortical architecture (e.g., prediction from genetically-derived [Fuzzy-clusters] versus gyral-based [Desikan] and sulcal- and gyral-based [Destrieux] anatomically-derived cortical ROIs). Furthermore, the Destrieux ROIs tend to be generally smaller than several of the Desikan and Fuzzy-clusters ROIs. In addition to gyral-based ROIs, including variables of small sulcal-based ROIs can help to enhance precision, especially considering that a substantial proportion of the cortical surface is sulcal. Neuroimaging data including 3D T1- and T2-weighted images were collected using Siemens Prisma, General Electric 750 and Phillips scanners with 3-Tesla systems. The ABCD Study® Data Analysis and Informatics Center (DAIC) implemented a quality control (QC) pipeline for MRI measures before and after processing the images. The DAIC processes, curates and shares the data using automated (e.g., signal-to-noise ratio), manual procedures (e.g., visual assessment of artifacts), and both approaches (e.g., automated feedback between workstations and staff; for more QC pipeline details, see Hagler Jr et al. 2019). Traveling quality assurance personnel receive the full test battery and MRI scans annually at each ABCD Study® site to harmonize the assessments. Brain volume sMRI variables (300) with datapoints below acceptable QC scores (6.8% of datapoint with scores < 600, based on the number of frames with framewise displacement < 0.2 mm during MRI scans; Dosenbach et al. 2017; Hagler Jr et al. 2019) were excluded from these analyses, coding them as missing. Data based on the other measures of the same subject were retained.

### Statistical analyses

#### TEs, PTSDsx and brain-imaging variables determination

Full information item factor analysis was applied to investigate the covariance structure of the 17 TEs and the 39 PTSDsx binary items that correspond to DSM-5 PTSD diagnosis. Single-factor models best accounted for the covariance structure of TEs (factor-loadings range = 0.46–0.96) and of PTSDsx (factor-loadings range = 0.40–0.97), explaining 57.9% and 68.5% of their variance, respectively. Item response theory (IRT) models were fitted separately to the TEs items, and PTSDsx data (see Supplementary Information 2 Figs. S1, S2, S3, S4, S5, S6). All the TEs items only reached 50% probability of responding positively when the liability was greater than the population mean. The probability of responding ‘yes’ to an item may be modeled as a sigmoidal curve, beginning at 0 for low values of liability, and increasing asymptotically to unity. The point on this curve where respondents have 50% probability of responding is known as its mean difficulty. A sum score of such items often has a hemi-modal distribution with many persons responding with zero symptoms. Fifteen TEs items had a 50% probability to be endorsed at two standard deviations (SDs) or more than the mean. Factor and IRT analyses supported the single-factor model for TEs data for children of this age range. In adult samples, a two-factor system for TEs is usually seen, where IPT is significantly associated with PTSD, while non-IPT events are not (Luthra et al. 2009). All the items corresponding to PTSDsx had 50% probability to be endorsed above 1.8 SDs on the θ axis. With the exception of four out of 39 PTSDsx items, all had a 50% probability to be endorsed at or above 3.9 SDs. Of the four items, one was part of criterion A (i.e., TEs; *SD* = 1.84) and the other three were part of the remaining criteria for PTSD diagnosis (*SDs* = 2.46–3.84). TEs and PTSDsx count variables based on raw binary items were skewed, with 97.08% of subjects endorsing from zero to two TEs (TEs range = 0–17), and 94.83% endorsing from zero to three PTSDsx (PTSDsx range = 0–20). Based on the full information item factor analysis and IRT models, one ordinal sum-score level variable was created for TEs (0–2) and another for PTSDsx (1–4). Unfortunately, treating the variables as ordinal in a multilevel context such as the one in this study (see *Phenotypic associations, and genetic and environmental variance decomposition* section) is impractical due to the curse of dimensionality.

Past TEs and current PTSDsx sum-score level variables had the fixed effects of age, sex and race/ethnicity removed by regression and these residuals were standardized. TEs and PTSDsx resulted in quasi-continuous variables after this process (TEs: N = 10,673, mean = 0, SD = 1, median =  − 0.6, min =  − 1.04, max = 2.68, skew = 1.13, kurtosis = 0.07; PTSDsx: N = 3794, mean = 0, SD = 1, median =  − 0.59, min =  − 1.28, max = 3.43, skew = 1.08, kurtosis = 0.53; see Supplementary Information 2, Figs. S7, S8). Maximum likelihood estimation and structural equation modeling (SEM), both used in this study, have shown that issues may arise while working with non-normally distributed data when values of skewness and kurtosis approach 2 and 7, respectively (Finney and DiStefano 2006), which it is not the case for the residualized and standardized TEs and PTSDsx variables. Nevertheless, the TEs and PTSDsx variables were also normalized in order to compare their parameter estimates with those of non-normal data of the same variables under the model proposed in this study. The volume of brain ROIs variables had the fixed effects of age, sex and race/ethnicity removed by regression as well, in addition to the fixed effects of type of scanner for all brain volume variables, total subcortical volume for subcortical ROIs and total cortical volume for the cortical parcellations. After the regression, the residuals from the brain volume variables were also standardized. Outlier datapoints surpassing + / − 4 SDs were coded as missing, subjects and their data from the other measures remained. The brain volumetric variables (*N* = 10,956 to 11,065) were continuous and resulted in 46.33% normally distributed (19 of the 42 subcortical, D = 0.006–0.013, p > 0.05; 120 of the 258 cortical, D = 0.004–0.013, p > 0.05; where a p < 0.05 indicates that a variable differs from a normal distribution under the Kolmogorov–Smirnov test Massey Jr 1951) with the remaining variables showing small departures from normality (see Supplementary Information 1c and 1d). Maximum likelihood estimation is generally robust to cases with these small departures (Benson and Fleishman 1994). All the volumetric brain-imaging phenotypes were included for variable-selection under an agnostic view, via regularization.

#### Regularization

Regularization methods integrate mathematical, statistical and machine learning processes to prevent model overfitting, to find an equilibrium in reducing variance without significantly increasing bias, and to select variables while favoring parsimony (Zou and Hastie 2005). Due to the numerous brain variables of volume in this study, regularization methods are well suited for selecting the most influential variables in this model while preventing overfitting. These methods are useful to process and learn from data that otherwise would be time and resource consuming or difficult to compute using explicit algorithms. The program or fitted model learns from the data and its experience, with the goal of improving its predictive performance in a task (Goodfellow et al. 2016; Mitchell 1997) rather than from explicit rules. Machine learning methods, and pertinent to this study, regularization, provide an agnostic approach to fitting models containing many variables.

Regularization techniques based on methods such as Ridge regression (Hoerl and Kennard 1970) and the least absolute shrinkage and selection operator (LASSO; Tibshirani 1996) add a penalty parameter λ to the penalty term (*L*^2^ parameter norm penalty for Ridge regularization, and *L*^1^ for LASSO) of the function in linear models to shrink the coefficients. As λ increases, the level of shrinkage of the coefficients increases. Of these two methods, LASSO is the only one able to shrink coefficients to zero, making possible to also select variables and produce sparse models. The penalty term of the function for the Ridge estimates includes the magnitude of the squared coefficients (Hastie et al. 2009) and reducing the residual sum of squares:

1 $${\widehat{\upbeta }}^{\mathrm{ridge}}=\mathrm{argmin}\left\{\sum_{\mathrm{i}=1}^{\mathrm{N}}{\left({\mathrm{y}}_{\mathrm{i}}- {\upbeta }_{0}- \sum_{\mathrm{j}=1}^{\mathrm{p}}{\mathrm{x}}_{\mathrm{ij}}{\upbeta }_{\mathrm{j}}\right)}^{2}+ \lambda \sum_{\mathrm{j}=1}^{\mathrm{p}}{{\upbeta }_{\mathrm{j}}}^{2}\right\}$$

whereas in LASSO (method penalizing the least squares), the absolute magnitude of the coefficients is used in the penalty term (Hastie et al. 2009)

2 $${\widehat{\upbeta }}^{\mathrm{lasso}}=\mathrm{argmin}\left\{\sum_{\mathrm{i}=1}^{\mathrm{N}}{\left({\mathrm{y}}_{\mathrm{i}}- {\upbeta }_{0}- \sum_{\mathrm{j}=1}^{\mathrm{p}}{\mathrm{x}}_{\mathrm{ij}}{\upbeta }_{\mathrm{j}}\right)}^{2}+ \lambda \sum_{\mathrm{j}=1}^{\mathrm{p}}\left|{\upbeta }_{\mathrm{j}}\right|\right\}.$$

Another regularization method is the elastic net (EN). EN favors sparse models by shrinking coefficients (albeit, not necessarily to zero) and allows for variable selection similar to LASSO. Nevertheless, EN can detect coefficients product of correlated variables allowing grouping effects, selecting or eliminating them, and preventing extreme shrinkage of correlated coefficients that did not reach the highest estimate in their group (Zou and Hastie 2005). In addition to λ, EN includes a tuning parameter α in the penalty term,

3 $$\lambda \sum_{\mathrm{j}=1}^{\mathrm{p}}\left(\mathrm{\alpha }{\upbeta }_{j}^{2}\right)+\left(1-\mathrm{\alpha }\right)\left|{\upbeta }_{\mathrm{j}}\right|,$$

setting EN as a hybrid between Ridge (α = 0) and LASSO (α = 1) regularization (Zou and Hastie 2005).

Due to the large number of brain phenotypes (300) in this study, cross-validation via k-fold subsampling (k = 10) using glmnet (Friedman et al. 2010), a package of the statistical programming language R (R Core Team 2019), was used for determining the α and λ parameters. Variables of the subcortical and cortical sets were fitted separately during cross-validation. A sequence of α values (between 0 and 1, with 0.05 increments) was created for these fits. The program set a sequence of λ values based on the data automatically for each k-fold during cross-validation. If the proportion of the null deviance explained does not change much across λ values, the program finishes and returns the minimum and maximum λ values. These two values restrict the fit between the smallest mean squared error (MSE) and one standard error from it, respectively. The λ values obtained from each cross-validation fit using different α values were inspected and those with the smallest MSE range were selected. These parameters were then used for fitting the models identifying the variables of brain volumes best explaining their associations with the indirect effects of TEs on PTSDsx. EN-regularization and SEM were used for selecting parsimonious models identifying the maximum number of brain-volume variables explaining the data while maintaining low MSE.

Several of the variables in this study were moderately to highly correlated (e.g., correlations of contralateral homologue ROIs: genetic = 0.75–0.99, phenotypic = 0.33–0.97), including some of the EN-identified brain ROIs, TEs and PTSDsx (see the Appendix, Figs. A1, A2, A3, A4, A5, A6). To account for potential bias from the non-independent correlated variables and for multiple testing, the eigenvalues of the brain-imaging variables correlation matrices are used to adjust the number of effective tests (Li and Ji 2005).

#### Phenotypic associations, and genetic and environmental variance decomposition

**Fig. 1:**
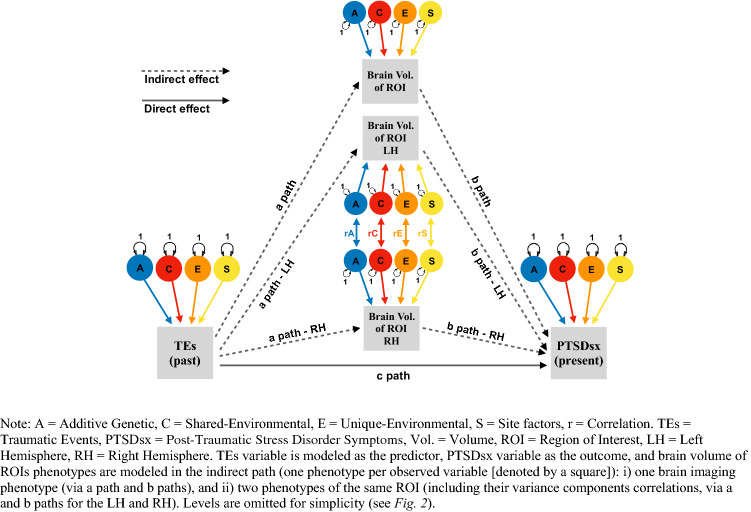
Multilevel mediation structural equation model

**Fig. 2:**
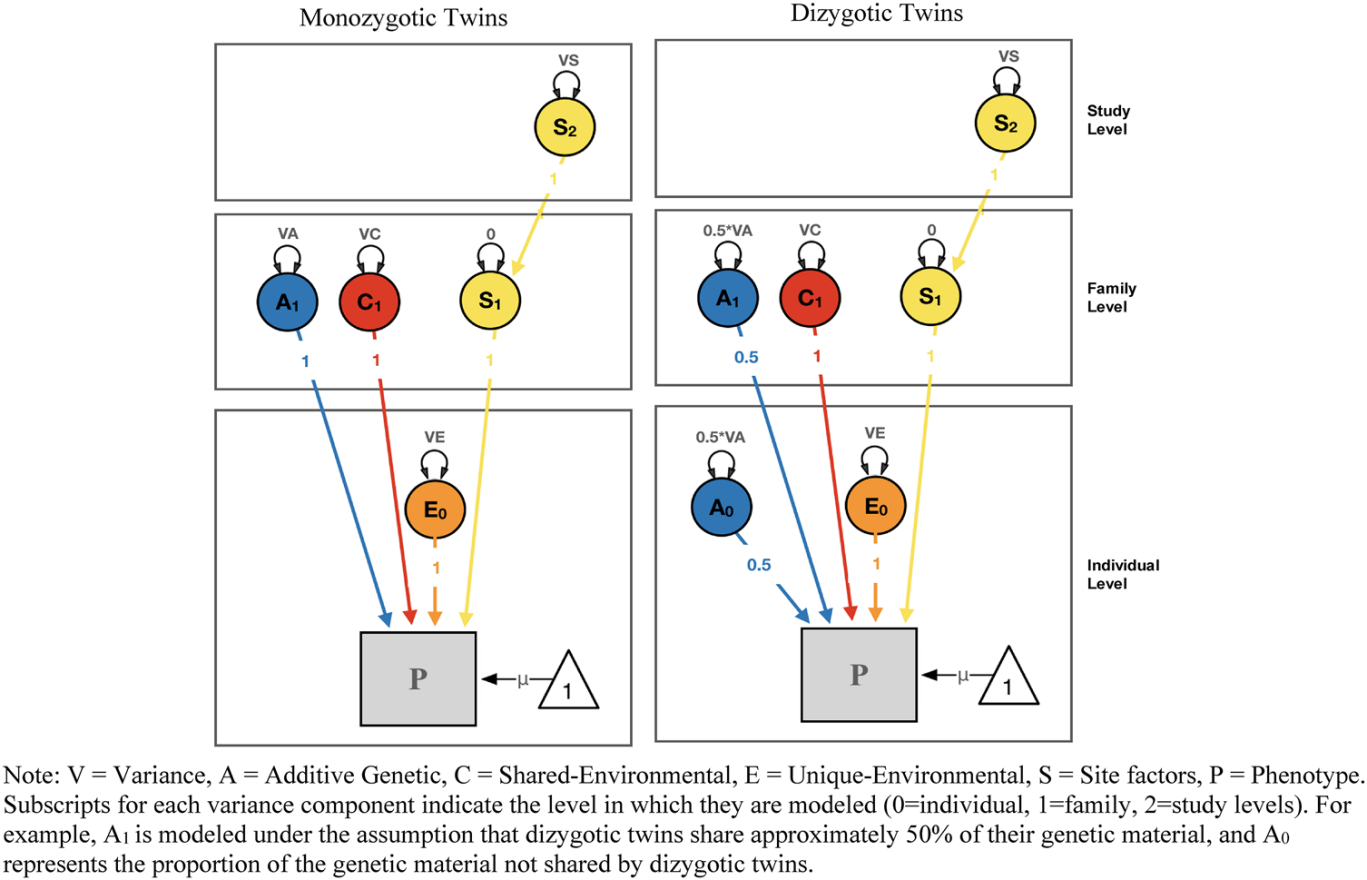
Multilevel variance components modeling

We based our multilevel structural equation model on a mediation model design, assessing the strength of the effects of TEs on PTSDsx through a direct c-path, and an indirect path (product of a- and b-path) via the neuroimaging phenotypes (see Figs. 1, 2). We fitted two EN-regularized models under this design: (i) subcortical ROIs using a minimum λ value of 0.0376, maximum λ value of 0.2222, and α parameter of 0.80; and (ii) cortical ROIs of Destrieux parcellation using a minimum λ value of 0.1783, maximum λ value of 0.97, and α parameter of 0.90; cortical Desikan and Fuzzy-clusters parcellations ROIs using a minimum λ value of 0.374, maximum λ value of 0.97, and α parameter of 0.35. The EN-selected brain ROIs were based on the highest estimates of the indirect effect paths, and on the number of variables restricted by the maximum MSE increase allowed. The contributions of additive genetic (A), common- (C), unique-environmental (E; including measurement error) and data-collection site (S) influencing the liability for past TEs, the volumes of EN-selected brain ROIs, and current PTSDsx were also estimated. OpenMx (a free and open source program for extended SEM; Neale et al. 2016; use version 2.19.6-31 for regularization functions) was used for fitting these models. The saturated model included the four estimated variance components (A, C, E and S) and means for each variable, covariance among contralateral brain ROIs, direct effect (c-path from TEs to PTSDsx, and reverse c-path from PTSDsx to TEs), and indirect effects via the volumes of EN-selected brain ROIs (a- and b-path from TEs to PTSDsx, and the reverse a- and b-path; see Fig. S9 on Supplementary Information 2). We then fitted three nested models constraining to zero: (i) the reverse direct c-path, (ii) the indirect reverse a- and b-path only, and (iii) the reverse direct c-path, and reverse indirect a- and b-path (leaving the unidirectional direct and indirect paths from TEs to PTSDsx in the model). Models were compared and selected based on their relative goodness-of-fit using likelihood ratio tests. Fit and parsimony were assessed using the twice-negative-log-likelihood (− 2lnL) statistic, and Akaike’s information criterion (AIC; Akaike 1998), respectively. The AIC combines goodness of fit based on − 2lnL estimates, with a penalty for less parsimonious models.

A variance components (A, C, E and S) model using only the twin sample was fitted for TEs, PTSDsx and the EN-selected brain volumes ROIs, in order to compare the parameter estimates with those from the full sample.

## Results

The analyses assessed the direct effects of TEs on PTSDsx, their indirect effects via the volumes of brain ROIs, and the genetic and environmental variation of these phenotypes.

### Cross-validation via EN-regularization and data reduction

The range of λ values (*subcortical* = 0.0376–0.2222, *cortical* = 0.1783–0.97) that restricted the fit within one standard error (SE) of maximum increase of MSE, identified five subcortical, one global, and nine cortical volumetric ROIs potentially associating with the indirect effects of TEs on PTSDsx. Gray matter volume, the left hemisphere (LH) of the cerebellar cortex, and the right lateral ventricle (all subcortical ROIs) were negatively associated with both TEs and PTSDsx. The left and right caudate nuclei (also subcortical ROIs) and cerebral white matter (WM) volume in the LH were positively associated with both TEs and PTSDsx. The supramarginal gyrus of the inferior parietal lobe in the LH, anterior transverse collateral sulcus in the LH, and the inferior temporal sulcus in the right hemisphere (RH; all cortical ROIs) were negatively associated with both TEs and PTSDsx. The anteromedial temporal, and dorsomedial frontal cortices (also cortical ROIs), both in the RH, were positively associated with TEs and negatively associated with PTSDsx. The medial-orbitofrontal cortex, the anterior part of the cingulate gyrus and sulcus, and the subcallosal gyrus, all in the LH, and the occipital lobe in the RH (all cortical ROIs) were positively associated with both TEs and PTSDsx.

### Goodness of fit, parsimony and model selection

Consistently across subcortical and cortical fits, the models with more parameters than the bidirectional c-path model [biDir; with unidirectional (uniDir) paths from TEs to PTSDsx directly and indirectly via the volumes of brain ROIs, and from PTSDsx to TEs] did not reach a solution, or the estimates SEs were large. The uniDir-reversed cortical model (with unidirectional paths from PTSDsx to TEs, and indirectly via the volumes of brain ROIs) reached a solution; however, some of the estimates and SEs were notably large. The uniDir model, estimating (i) direct c-, and indirect a- and b-paths from TEs to PTSDsx via the volumes of EN-selected brain ROIs, and (ii) additive-genetic, shared-, unique-environmental and site factors influencing the phenotypes, showed the most parsimonious fit to the data in the subcortical and cortical models. We compared the uniDir model to the more complex biDir c-path model separately for the subcortical and cortical fits. In both cases, the uniDir model was selected via likelihood ratio tests. Although the biDir subcortical model fitted better than the uniDir, the latter was more parsimonious without significantly worsening the fit (biDir: − *2lnL* = 198,018.06, *AIC* = 42,126.06; uniDir*: − 2lnL* = 198,018.37, *AIC* = 42,124.37, *Δχ*^2^(1) = 0.303; see Table 1). The cortical biDir and uniDir models fit the data equally well. However, the uniDir cortical model was selected due to its greater parsimony (biDir: − *2lnL* = 309,610.2, *AIC* = 87,564.197; uniDir*: − 2lnL* = 309,610.2, *AIC* = 87,562.197, *Δχ*^2^(1) = 2.84 × 10^−8^; see Table 1).

**Table 1:** Models comparison

	Base	Comparison	ep	− 2LL	df	AIC	diffLL	diffdf	p
1S	biDir c-path (S)		58	198,018.06	77,946	42,126.06			
2S	biDir c-path (S)	uniDir Reversed (S)	57	198,075.50	77,947	42,181.53	57.47	1	0
3S	biDir c-path (S)	uniDir (S)	57	198,018.37	77,947	42,124.37	0.30	1	0.58
1C	biDir c-path (C)		75	309,610.20	111,023	87,564.197			
2C	biDir c-path (C)	uniDir (C)	74	309,610.20	111,024	87,562.197	0.0000	1	1.00

The Reversed model includes a ‘c’ direct, and ‘a’ and ‘b’ indirect paths from PTSDsx to TEs via the volumes of EN-selected brain ROIs

*S* subcortical, *C* cortical, *biDir c-path* bidirectional ‘c’ path model, *uniDir* unidirectional model, *ep* estimated parameters, *LL* log-likelihood, *AIC* Akaike’s information criterion, *PTSDsx* post-traumatic stress disorder symptoms, *TEs* traumatic events

### Direct effects of TEs on PTSDsx and their indirect effects via the volumes of brain ROIs

The direct effect (c-path on Fig. 1) of past TEs on current PTSDsx was high (*subcortical* = 0.918, *cortical* = 0.914). Cerebral WM in the LH (indirect effect: 0.00037), the left caudate nucleus (indirect effect: 0.00015) and right lateral ventricle (indirect effect: 0.00007; both subcortical ROIs), and the anterior transverse collateral sulcus in the LH (indirect effect: 0.0003; cortical ROI) were the only EN-selected ROIs with 95% confidence intervals (CIs) lower bounds greater than zero, but their indirect effects were miniscule. Table 2 shows the indirect effects estimates and CIs of these brain ROIs. The a- and b-paths (TEs → ROIs and ROIs → PTSDsx, respectively) were positive for cerebral WM in the LH (*a* = 0.014; *b* = 0.027) and left caudate nucleus (*a* = 0.014; *b* = 0.010), but negative for the right lateral ventricle (*a* =  − 0.009; *b* =  − 0.008) and anterior transverse collateral sulcus (*a* =  − 0.015; *b* =  − 0.021).

**Table 2:** Indirect effects estimates and 95% confidence intervals of elastic-net selected subcortical and cortical ROIs from TEs on PTSDsx

	Lower bound	Estimate	Upper bound
Cerebral WM in the LH	0.00035	0.00037	0.00039
Caudate nucleus LH (S)	0.00013	0.00015	0.00017
Lateral ventricle RH (S)	0.00007	0.00007	0.00007
Anterior transverse collateral sulcus LH (C)	0.00004	0.00030	0.00110

The table includes estimates and CIs only of those EN-selected brain ROIs not containing zero

*ROIs* regions of interest, *TEs* traumatic events, *PTSDsx* post-traumatic stress disorder symptoms, *WM* white matter, *LH* left hemisphere, *RH* right hemisphere, *S* subcortical, *C* cortical

### Twin correlations

Twin correlations of TEs, PTSDsx, and the volumes of EN-selected ROIs were estimated by maximum likelihood using a saturated model (see Table 3). The cross-twin correlations for TEs and PTSDsx in DZ twins (0.77 and 0.66, respectively) are considerably higher than half of the MZ correlations (0.82 and 0.75, respectively). A similar pattern arises for the cross-twin cross-trait correlations, where those of MZ pairs (*TEs-twin1[T1]–PTSDsx-twin2[T2]* = 0.69 and *TEsT2–PTSDsxT1* = 0.67) are slightly higher than those of DZ pairs (*TEsT1–PTSDsxT2* = 0.51 and *TEsT2–PTSDsxT1* = 0.52). For EN-selected subcortical phenotypes and cerebral WM in the LH, twin correlations for MZ twins are moderate to high (0.66–0.91) and twice or greater than the DZs’ correlations (0.24–0.50) of the same phenotype, with one exception (left cerebellar cortex: *rMZ* = 0.91; *rDZ* = 0.50). MZ correlations (0.26–0.67) for EN-selected cortical phenotypes are greater than twice those of DZ twins (0.07–0.42) of the same phenotype, except for the occipital lobe in the RH (*rMZ* = 0.67; *rDZ* = 0.42). The cross-twin cross-trait correlations are low to moderate for MZ and DZ twins, besides those for the caudate nuclei in MZ twins (*CaudateRHT1–CaudateLHT2* and *CaudateLHT1–CaudateRHT2 rMZs* = 0.82).

**Table 3:** Twin correlations: MZ and DZ pairs

	TEs T1	PTSDsx T1	(S) Caudate LH T1	Cerebral WM LH T1	(S) Lat. Vent. RH T1	(C-Dest.) ATrCoS LH T1	TEs T2	PTSDsx T2	(S) Caudate LH T2	Cerebral WM LH T2	(S) Lat. Vent. RH T2	(C-Dest.) ATrCoS LH T2
TEs T1	**1**	**0.67** (0.56, 0.78)	** − 0.06** (− 0.16, 0.04)	**0.1** (0, 0.2)	− **0.02** (− 0.12, 0.08)	− **0.01** (− 0.11, 0.1)	**0.77** (0.73, 0.81)	**0.51** (0.36, 0.65)	**0.02** (− 0.08, 0.13)	− **0.03** (− 0.14, 0.07)	**0** (− 0.1, 0.11)	**0.1** (0, 0.2)
PTSDsx T1	**0.73** (0.62, 0.83)	**1**	**0.05** (− 0.15, 0.24)	**0.17** (− 0.02, 0.36)	− **0.06** (− 0.25, 0.13)	**0.01** (− 0.18, 0.21)	**0.52** (0.38, 0.66)	**0.66** (0.55, 0.77)	**0.08** (− 0.11, 0.27)	**0.07** (− 0.13, 0.26)	**0.07** (− 0.12, 0.26)	− **0.04** (− 0.23, 0.15)
(S) Caudate LH T1	**0.04** (− 0.07, 0.16)	**0.01** (− 0.2, 0.23)	**1**	**0** (− 0.1, 0.1)	**0.34** (0.25, 0.42)	**0** (− 0.1, 0.1)	− **0.01** (− 0.11, 0.09)	**0.09** (− 0.11, 0.28)	**0.41** (0.33, 0.5)	**0.01** (− 0.09, 0.11)	**0.04** (− 0.06, 0.14)	− **0.11** (− 0.21, − 0.01)
Cerebral WM LH T1	**0.07** (− 0.05, 0.18)	**0.19** (− 0.02, 0.39)	− **0.01** (− 0.12, 0.11)	**1**	**0.04** (− 0.06, 0.14)	**0.13** (0.03, 0.23)	**0.09** (− 0.02, 0.19)	**0.17** (− 0.02, 0.36)	− **0.05** (− 0.15, 0.05)	**0.45** (0.37, 0.53)	**0** (− 0.1, 0.1)	**0.01** (− 0.09, 0.11)
(S) Lat. Vent. RH T1	− **0.01** (− 0.12, 0.11)	**0.05** (− 0.17, 0.26)	**0.29** (0.18, 0.39)	− **0.01** (− 0.13, 0.1)	**1**	**0.11** (0.01, 0.21)	**0** (− 0.1, 0.11)	− **0.07** (− 0.26, 0.12)	**0.18** (0.09, 0.28)	**0.06** (− 0.04, 0.16)	**0.24** (0.14, 0.33)	**0.07** (− 0.03, 0.17)
(C-Dest.) ATrCoS LH T1	**0.01** (− 0.11, 0.12)	− **0.14** (− 0.35, 0.07)	− **0.03** (− 0.14, 0.08)	**0.09** (− 0.02, 0.2)	− **0.08** (− 0.19, 0.04)	**1**	**0.01** (− 0.09, 0.12)	**0.04** (− 0.16, 0.23)	**0.04** (− 0.06, 0.14)	**0** (− 0.1, 0.1)	**0.05** (− 0.05, 0.15)	**0.1** (0, 0.2)
TEs T2	**0.82** (0.78, 0.85)	**0.67** (0.55, 0.79)	− **0.04** (− 0.16, 0.07)	**0.05** (− 0.06, 0.17)	− **0.03** (− 0.15, 0.08)	**0.05** (− 0.07, 0.16)	**1**	**0.58** (0.45, 0.71)	**0.06** (− 0.04, 0.16)	− **0.03** (− 0.13, 0.07)	− **0.02** (− 0.12, 0.08)	**0.07** (− 0.03, 0.17)
PTSDsx T2	**0.69** (0.58, 0.8)	**0.75** (0.65, 0.84)	− **0.08** (− 0.3, 0.13)	**0.27** (0.08, 0.47)	− **0.02** (− 0.23, 0.19)	− **0.08** (− 0.29, 0.13)	**0.73** (0.63, 0.83)	**1**	**0.01** (− 0.18, 0.21)	**0.02** (− 0.18, 0.21)	− **0.01** (− 0.21, 0.18)	− **0.06** (− 0.25, 0.13)
(S) Caudate LH T2	**0** (− 0.11, 0.12)	− **0.06** (− 0.28, 0.15)	**0.84** (0.81, 0.88)	− **0.01** (− 0.13, 0.1)	**0.25** (0.14, 0.36)	− **0.04** (− 0.15, 0.08)	− **0.06** (− 0.17, 0.06)	− **0.07** (− 0.28, 0.15)	**1**	− **0.04** (− 0.14, 0.06)	**0.27** (0.17, 0.36)	**0.01** (− 0.09, 0.11)
Cerebral WM LH T2	**0.06** (− 0.05, 0.18)	**0.08** (− 0.13, 0.29)	− **0.02** (− 0.14, 0.09)	**0.9** (0.88, 0.92)	− **0.03** (− 0.14, 0.09)	**0.07** (− 0.04, 0.19)	**0.04** (− 0.07, 0.16)	**0.14** (− 0.06, 0.35)	− **0.03** (− 0.15, 0.08)	**1**	**0.02** (− 0.08, 0.12)	**0.13** (0.03, 0.23)
(S) Lat. Vent. RH T2	**0.02** (− 0.09, 0.14)	**0.04** (− 0.18, 0.25)	**0.37** (0.27, 0.47)	− **0.01** (− 0.13, 0.1)	**0.66** (0.6, 0.73)	− **0.12** (− 0.23, 0)	**0** (− 0.12, 0.11)	− **0.02** (− 0.23, 0.2)	**0.41** (0.31, 0.5)	− **0.02** (− 0.14, 0.09)	**1**	**0.1** (0, 0.2)
(C-Dest.) ATrCoS LH T2	**0.04** (− 0.07, 0.16)	− **0.08** (− 0.29, 0.14)	− **0.04** (− 0.15, 0.08)	**0.06** (− 0.05, 0.18)	− **0.05** (− 0.17, 0.06)	**0.26** (0.16, 0.37)	**0.02** (− 0.1, 0.13)	− **0.08** (− 0.3, 0.13)	− **0.05** (− 0.17, 0.06)	**0.04** (− 0.07, 0.15)	− **0.03** (− 0.14, 0.08)	**1**

Correlation estimates are shown in bold

Twin correlations for TEs, PTSDsx, and EN-selected brain ROIs of volume not containing zero in their CIs are included in the table. MZ correlations are shown below the diagonal, DZ correlations are shown above the diagonal. Confidence intervals (CIs; 95%) are given between parentheses

*MZ* monozygotic, *DZ* dizygotic, *TEs* traumatic events, *PTSDsx* post-traumatic stress disorder symptoms, *T1* Twin 1, *T2* Twin 2, *S* subcortical, *C* cortical, *WM* white matter, *LH* left hemisphere, *RH* right hemisphere, *Lat.* lateral, *Vent.* Ventricle, *Dest.* Destrieux, *ATrCoS* anterior transverse collateral sulcus

### Genetic and environmental contributions to phenotypic variance

Multilevel analyses show (see Table 4) that the variability in past TEs was explained mostly by common-environmental factors (61.63%) with modest influence of additive genetic factors (23.36%) and a small contribution of unique-environmental factors (15.13%). Residual components for current PTSDsx accounted for 61.34% of its variance (*res-A* = 20.89%, *res-C* = 18.57%, *res-E* = 21.88%). Additive genetic factors associate with most of the variance in the EN-selected cerebral WM in the LH (57.35%) and subcortical ROIs (52.38–61.75%; subcortical gray matter = 61.75%, cerebellar cortex LH = 53.56%, caudate nucleus LH = 54.43%, caudate nucleus RH = 52.38%) except for the lateral ventricle RH (41.23%), where more of the variability was accounted for by unique-environmental factors (43.08%). Most of the phenotypic variation in the volumes of cortical ROIs was accounted for by additive genetic factors in the occipital lobe (52.81%), anteromedial temporal (46.36%) and dorsomedial frontal cortices (53.28%), all in the RH and belonging to the Fuzzy-clusters parcellation. Additive genetic factors explained a lower proportion of the variation in the remaining six EN-selected cortical ROIs (medial–orbitofrontal cortex LH = 30.17%, anterior cingulate gyrus and sulcus LH = 28.95%, inferior temporal sulcus RH = 23.83%, supramarginal gyrus of the inferior parietal lobe LH = 20.27%, subcallosal gyrus LH = 18.57%, and the anterior transverse collateral sulcus LH = 15.11%). Common-environmental factors had a small to moderate influence (4.48–36.39%) on the cortical ROIs, notably higher for the right occipital lobe (33.87%), anteromedial temporal (36.39%) and dorsomedial frontal (29.82%) cortices on the RH. Unique-environmental factors explained most of the variation (56.72–74.76%) in volumes of the medial–orbitofrontal cortex LH, anterior cingulate gyrus and sulcus LH, supramarginal gyrus of the inferior parietal lobe LH, subcallosal gyrus LH, anterior transverse collateral sulcus, and the inferior temporal sulcus RH. Site variance was small for TEs, PTSDsx (both < 1.00%), and neuroimaging phenotypes (0.20–0.87%), with that of the dorsomedial frontal cortex slightly higher (1.97%) than the other brain ROIs. Considering the twin-sample fit (see Table A1 in the Appendix), the influence of additive genetic factors increased by an average of 24.98% compared to the full-sample fit, with the exception of TEs, PTSDsx and the occipital lobe*.* The variation of volumes of brain ROIs phenotypes explained by shared-environmental factors decreased by an average of 25.1% from the full sample to the twins-only fit, whereas it increased for TEs by 12.8% and 8.3% for PTSDsx*.* Changes in the estimates of unique-environmental factors from the full- to the twin-sample fit remained within < 6% for all phenotypes except for the occipital lobe, dorsomedial frontal cortex, anteromedial temporal cortex and lateral ventricle, all in the RH. Changes in the site component from both fits remained below 1.3%.

**Table 4:** Variance components estimates (TEs, EN-selected ROIs and PTSDsx) with 95% CIs: full sample

	**VA**	LB	UB	**VC**	LB	UB	**VE**	LB	UB	**VS**	LB	UB
TEs	**0.23**	0.19	0.27	**0.62**	0.57	0.66	**0.15**	0.13	0.17	**0.01**	0.001	0.01
(S) Caudate nucleus LH	**0.54**	0.48	0.61	**0.29**	0.23	0.34	**0.17**	0.14	0.20	**0.001**	− 0.001	0.003
(S) Caudate nucleus RH	**0.52**	0.45	0.59	**0.28**	0.22	0.33	**0.20**	0.17	0.23	**0.002**	− 0.001	0.004
(S) Cerebellar cortex LH	**0.54**	0.48	0.59	**0.37**	0.32	0.42	**0.10**	0.08	0.11	**0.003**	− 0.0003	0.01
Cerebral WM LH	**0.57**	0.51	0.63	**0.31**	0.26	0.37	**0.11**	0.10	0.13	**0.004**	− 0.0001	0.01
(S) Lat. Vent. RH	**0.41**	0.31	0.51	**0.15**	0.09	0.21	**0.43**	0.37	0.49	**0.002**	− 0.001	0.005
(S) Gray matter	**0.62**	0.55	0.68	**0.27**	0.21	0.33	**0.11**	0.09	0.13	**0.01**	0.002	0.01
(C-Desik.) Medial of cortex LH	**0.30**	0.18	0.42	**0.04**	− 0.02	0.11	**0.65**	0.56	0.73	**0.01**	0.002	0.02
(C-Dest.) ACgG/S LH	**0.29**	0.18	0.40	**0.14**	0.07	0.20	**0.57**	0.49	0.64	**0.01**	0.001	0.01
(C-Dest.) InfSuPG LH	**0.20**	0.09	0.32	**0.08**	0.02	0.15	**0.71**	0.63	0.80	**0.004**	0.0001	0.01
(C-Dest.) SbCaG LH	**0.19**	0.06	0.31	**0.10**	0.04	0.17	**0.71**	0.61	0.80	**0.002**	− 0.0005	0.01
(C-Dest.) ATrCoS LH	**0.15**	0.03	0.27	**0.10**	0.03	0.17	**0.75**	0.66	0.84	**0.003**	− 0.0003	0.01
(C-Dest.) InfTS RH	**0.24**	0.12	0.36	**0.05**	− 0.01	0.12	**0.71**	0.62	0.79	**0.003**	− 0.0002	0.01
(C-Fuzzy) AMT cortex RH	**0.46**	0.40	0.53	**0.36**	0.31	0.42	**0.17**	0.14	0.20	**0.01**	0.001	0.01
(C-Fuzzy) DMF cortex RH	**0.53**	0.47	0.60	**0.30**	0.24	0.36	**0.16**	0.13	0.18	**0.02**	0.01	0.03
(C-Fuzzy) Occipital lobe RH	**0.53**	0.47	0.59	**0.34**	0.28	0.39	**0.13**	0.11	0.15	**0.01**	0.002	0.01
PTSDsx	**0.21**	0.09	0.32	**0.18**	0.10	0.27	**0.22**	0.15	0.28	**0.005**	− 0.001	0.01

Variance components estimates are shown in bold

*TEs* traumatic events, *EN* elastic net, *ROIs* regions of interest, *PTSDsx* post-traumatic stress disorder symptoms, *CIs* confidence intervals, *VA* variance explained by additive genetic factors, *VC* variance explained by common-environmental factors, *VE* variance explained by unique-environmental factors, *VS* variance explained by site factors, *LB* CIs lower bound, *UB* CIs upper bound, *S* subcortical, *C* cortical, *WM* white matter, *LH* left hemisphere, *RH* right hemisphere, *Lat.* lateral, *Vent.* Ventricle, *OF* orbitofrontal, *ACgG/S* anterior part of the cingulate gyrus and sulcus, *InfSuPG* supramarginal gyrus of the inferior parietal lobe, *SbCaG* subcallosal gyrus, *InfTS* inferior temporal sulcus, *AMT* anteromedial temporal, *DMT* dorsomedial frontal, *ATrCoS* anterior transverse collateral sulcus, *Desik.* Desikan, *Dest.* Destrieux

The differences between the paths parameter estimates from our model fits compared to those using normalized TEs and PTSDsx data were very small (< 0.0004). Moreover, their variance components estimates changed less than 1% between these fits with only two exceptions (TEs VA: from 23.4 to 27.3%; TEs VC from 61.6 to 57.4%).

## Discussion

This study estimated the direct influence of past TEs on current PTSDsx, and their indirect effects through the volumes of 300 brain ROIs in children 9–11 years old using EN-regularization, an agnostic machine learning approach. As hypothesized, a greater number of past TEs was strongly associated with higher current PTSDsx in children. Conversely, and contrary to our second hypothesis, there is negligible influence from the volumes of brain ROIs on the indirect effects of TEs on PTSDsx at this age. Furthermore, we estimated the genetic and environmental variance components explaining the variability in past TEs, brain volumes and current PTSDsx. Environmental factors explained most of the variance in TEs, and of the volumes of six EN-selected cortical ROIs, and most of the residual environmental variance in PTSDsx. For EN-selected brain ROIs, additive genetic factors accounted for most of the variation in volumes of three cortical phenotypes, cerebral WM in the LH, and all subcortical ROIs with the exception of the right lateral ventricle. A very small proportion of the variance was accounted for by factors from study sites.

The EN-selected brain ROIs with the highest influence on the indirect path from TEs on PTSDsx were the left caudate nucleus, right lateral ventricle, cerebral WM in the LH, and the left anterior transverse collateral sulcus. The four brain-imaging phenotypes had miniscule effects; however, they were the only EN-selected ROIs not containing zero in their CIs. Possibly, the aggregated small effects of many volumetric brain ROIs influence the outcome, and if the effects exist, their influence may increase in later stages of development. As children age, the likelihood of experiencing more TEs and a greater proportion of IPT will increase, potentially uncovering larger direct and indirect effects. Moreover, other brain-imaging phenotypes (e.g., activation measured via functional MRI) could also inform about the indirect influence of TEs on PTSDsx. Furthermore, while larger associations were observed between TEs and brain measures, and between brain measures and PTSDsx, the two do not seem to coordinate to provide evidence of the indirect effects under the mediation model design at this age. Brain ROIs volumes may associate differently with TEs than with PTSDsx in 9–11 year-olds.

EN-selected subcortical ROIs including those with the highest indirect effect estimates (i.e., volumes of the left caudate nucleus, and right lateral ventricle), and the volume of cerebral WM in the LH, are consistent with ROIs associated with PTSD in prior work (Clausen et al. 2019; Cohen et al. 2006; De Bellis et al. 2002; Herringa et al. 2012). However, the direction of their relationship with TEs (a-paths) and PTSDsx (b-paths) differs from those found in prior research assessing trauma and PTSDsx during adulthood (Cohen et al. 2006; Herringa et al. 2012) and retrospective childhood trauma (Clausen et al. 2019). These three ROIs are associated with processes relevant to PTSD. For example, the caudate nucleus is linked to reward anticipation/response, and anhedonia processes linked to PTSD and other disorders (e.g., major depressive disorder; Nawijn et al. 2015). Nevertheless, the volume of this ROI has been associated differently with features of PTSD depending on trauma type, developmental stage and sample type (e.g., civilian versus combat-exposed; Cohen et al. 2006; Looi et al. 2009). The volumes of the lateral ventricles relate directly to subcortical tissue volume, and to cerebral-spinal fluid (CSF) volume. The relation to CSF potentially highlights processes in which the CSF is involved in the brain (e.g., circulation of nutrients, waste removal), and associations with PTSD, for example in combat-exposed samples with history of early trauma (Woodward et al. 2007). However, the CSF volume was not selected via EN influencing the indirect effects of TEs on PTSDsx. The EN selection of a cavity ROI such as the right lateral ventricle is perhaps more closely associated with the volumes of neighboring ROIs such as the caudate nuclei. Cerebral WM relates to cortical–subcortical connectivity, behavioral and cognitive functions. Different volumes and integrity of cerebral white matter have been associated with PTSD. However, larger or smaller volumes vary in their association with this phenotype depending if traumas were experienced during childhood or if the onset of PTSD diagnosis was during adulthood (e.g., Daniels et al. 2013). The anterior transverse collateral sulcus is part of the default mode network, associated with introspection, rumination and social cognition (Li et al. 2014), and has been linked to early trauma in adults (Coley et al. 2021). Although the EN-selected ROIs not containing zero in their CIs have been associated with PTSD, trauma and PTSD features, their effects on the indirect influence of TEs on PTSDsx were negligible. Assessing the effects of different types of trauma (e.g., IPT, non-IPT) on PTSDsx by sex and longitudinally may render more insights from a developmental perspective, and represents a natural next step in this line of research.

Other regions repeatedly associated with TEs and PTSD, such as the hippocampus and the amygdala (Kitayama et al. 2005; Logue et al. 2018; Morey et al. 2016), were not identified via EN influencing the full indirect path (a * b) from past TEs to subsequent PTSDsx for children of this age. A few brain ROIs not selected via EN, but associated with TEs and PTSDsx in prior work (e.g., right hippocampus; Gilbertson et al. 2002; Thomaes et al. 2010), reached higher estimates on their independent a- or b-path compared to those of EN-selected ROIs with full indirect effects not containing zero in their CIs. However, the effects of the non-selected ROIs via EN were reduced when taking in account their full path. For example, two of the strongest associations with TEs (a-path) were shown by the volume of the right hippocampus (*a* =  − 0.0198) and the right amygdala (*a* =  − 0.013). These associations are greater or close to those from the a-paths of EN-selected ROIs not containing zero in their CIs. Nevertheless, the associations of the right hippocampus and the right amygdala with PTSDsx were smaller and positive (*b* = 0.0055, 0.0105; respectively) compared to these two ROIs’ associations with TEs. Although assessing the specific a- and b-paths was not an aim of this study, future analyses focusing on independent indirect paths could inform about possible specific relationships that volumetric brain ROIs may develop with TEs and PTSDsx differently. Perhaps the volumes of certain brain ROIs may associate with the effects of TEs on PTSDsx and their trauma-related cluster, while other ROIs associate to a higher degree with other PTSD clusters (e.g., reexperiencing). Our future directions include longitudinal modeling of these specific paths.

Additive genetic factors explained most of the variation in the three EN-selected Fuzzy-clusters ROIs, and in all the EN-selected volumes of subcortical ROIs with the exception of the right lateral ventricle. The results for the additive genetic factors influencing the volumes of brain imaging phenotypes are generally consistent with previous research (Afifi et al. 2010; Schmitt et al. 2007), with lower heritability estimates compared to adults (Brouwer et al. 2017). The higher influence of additive genetic factors on such subcortical and Fuzzy-clusters ROIs compared to those of the anatomically-based cortical volumetric phenotypes in children may infer greater environmental effects for the latter phenotypes. Furthermore, although A estimates range from low-moderate to high for children, these results may indicate that there is generally higher influence of environmental factors on brain MRI volumetric phenotypes during childhood compared to adulthood. Findings from the additive genetic factors influencing EN-selected Fuzzy-clusters ROIs can inform MRI research focusing on ROI activation, since this genetically-based atlas aligns with cortical functional segmentation. Higher neuroplasticity in children and environmental influences on certain ROIs (e.g., lateral ventricles, anatomically-based cortical ROIs), and greater variance of the non-twin subjects may drive lower heritability estimates for children at this age (Brouwer et al. 2017). Greater measurement error during MRI scanning (e.g., due to motion artifacts), may occur in children than adults (Blumenthal et al. 2002). Interestingly, environmental factors were the largest source of phenotypic variance, and particularly unique-environmental factors in the lateral ventricle RH, and in the following cortical ROIs: medial–orbitofrontal cortex LH, anterior cingulate gyrus and sulcus LH, supramarginal gyrus of the inferior parietal lobe LH, subcallosal gyrus LH, anterior transverse collateral sulcus, and the inferior temporal sulcus RH. Since error of measurement is included in E estimates, some small cortical ROIs (such as a few from the Destrieux and Desikan parcellations) might be more susceptible to error than subcortical ROIs, in addition to the contributions of unique-environmental factors.

Common-environmental factors were highly influential in explaining the exposure to TEs in children, showing higher estimates than in prior literature assessing trauma mostly in older non-combat cohorts (Stein et al. 2002). This indicates that the rearing environment may influence more the variation of TEs for children of this age than for adults. However, reports from parents about their children’s TEs experiences may overestimate the influence of common-environmental factors on this phenotype, due to variation in parents’ rating styles. Similarly, uncontrolled associations between age and the outcome could increase C estimates. Nevertheless, the effects of age on C were accounted for by regression. The effects of assortative mating would similarly mimic those of C, but estimates of marital resemblance for TEs, neuroimaging measures and PTSD are few. Those that exist for psychiatric phenotypes suggest low marital correlations (from − 0.05 to 0.21), making assortment unlikely to contribute substantially to variation (Maes et al. 1998). Additive genetic factors had a small influence on TEs variation in children, lower than that observed in adults for exposure to violent interpersonal trauma (Stein et al. 2002). Endorsement frequencies for the types of TEs assessed in our study (e.g., IPT, accidental, witnessing) varied. IPT items were endorsed at low frequencies, and witnessing and accidental TEs had a larger proportion of endorsement at this age. Low frequency response rate items usually correspond to greater measurement error, because an incorrect response or diagnosis would change the low response rate to a greater extent—consistent with the standard error of a binomial distribution. Prior work found that more variability in IPT is associated with additive genetic factors than with environmental factors, with the latter explaining most of the variability in non-IPT measures (Sartor et al. 2012; Stein et al. 2002). In addition, information curves for TEs items were skewed to an extreme location in the θ liability scale, where 15 out of 17 items discerned the endorsement to each with 50% probability at above 2 SDs from the mean. Most of these items were associated with direct threats or IPT to the children, which were fortunately rare. In contrast, the two items with the inflection points of their information curves below + 2 SDs were associated with learning about death and witnessing violence at home. High endorsement of non-IPT events might be driving the C estimates upwards. It is also possible that the variability explained by common-environmental factors in TEs in children is affected in part by the parental environment given to the twins and siblings.

After correcting for covariates, most of the variation in PTSDsx was explained by environmental factors. The influence of additive genetic factors in children here is lower than those for PTSD and PTSDsx found in twin studies of adult samples (Amstadter et al. 2012; Stein et al. 2002; Wolf et al. 2018) and higher than the SNP-based heritability estimates from molecular work (Nievergelt et al. 2019). In contrast to previous work assessing adults from non-combat samples, in which most and almost all of the variability in PTSDsx from environmental sources was accounted for by unique-environmental factors (Afifi et al. 2010; Stein et al. 2002; Wolf et al. 2018), the variation of PTSDsx in children of this age is influenced by common-environmental in addition to unique-environmental factors. Similar to the information curves from TEs, PTSDsx items were also skewed to an extreme location in the θ scale. Only four of 39 items reached 50% endorsement probability below + 3.9 SDs from the mean. These items were associated with learning about “the sudden and unexpected death of a loved one”, or with changes in mood and emotional reactions (e.g., behaviors qualifying as self-destructive or reckless, clinical distress, impairment in functioning) that can be readily displayed at home. Measurement of these traits in youth remains problematic, so their associations with risk factors and between relatives are likely to be eroded. Shared experiences at home, among family members and twins may increase the liability for experiencing PTSDsx moderated by genotypes. Although our study was not designed to detect gene–environment interaction (G × E), it might be possible that the type of TE may alter their expected influence on PTSDsx, based on specific genetic variants.

We compared the variance components estimates obtained from the full-sample fit with those from the twins-only sample to consider further interpretations of the genetic and environmental influences for the same phenotypes*.* This comparison is an additional resource rather than a study aim. With the exception of the right occipital lobe, TEs and PTSDsx, A estimates increased for all the phenotypes in the twin sample fit. These results also support those from the full sample and prior work (Christova et al. 2021; Patel et al. 2018; Schmitt et al. 2019) that generally, A estimates for cortical phenotypes tend to lower than those from subcortical ROIs (with a few exceptions). Shared-environmental factors estimates were negative for most of the brain-imaging phenotypes in the twins-only fit. Negative C estimates can infer the presence of non-additive genetic factors explaining phenotypic variation. Contrasting to the full-sample fit, the right occipital lobe is the only EN-selected phenotype with shared-environmental factors influencing over 9% of its variance in the twins-only fit. This may indicate higher neuroplasticity in the occipital lobe than in the other cortical regions, influenced by environmental stimuli at home or other common settings.

Additive genetic factors estimates for TEs and PTSDsx from the full sample were higher than those using the twins alone. Variance differences between twins and non-twins likely reduced the estimates of heritability. Nevertheless, the residual influences of shared- and unique-environmental factors became more similar in PTSDsx as opposed to in TEs, with shared-environmental factors driving most of the variance in TEs in both samples. This may support that, in contrast to adult samples, shared-environmental factors contribute to the variation in TEs and PTSDsx in children of this age, based mostly on non-IPT events. This result seems likely due to greater environmental sharing at this age. Potentially, G × E and differences in ascertainment for the non-twin and twin individuals may drive some of the differences in additive genetic and environmental contributions to these phenotypes.

Finally, for all phenotypes, the proportion of site variance in both samples was low and similar. These results attest to a high degree of QC across the ABCD Study® sites.

### Limitations

Findings from this study must take in account the following five potential limitations. First, measures of TEs and PTSDsx are skewed due to the low endorsement of at least one TE, and PTSDsx. This may have potentially biased the direct and indirect effects towards zero, increased the proportion of residual variance (E), and reduced statistical power to a certain degree. However, their skewness and kurtosis statistics were below what it is commonly seen as problematic (2 and 7, respectively) under SEM and maximum likelihood estimation (Finney and DiStefano 2006). Second, the assessment of past TEs was based on retrospective report from the parents, potentially affected by recall bias. Nevertheless, parents’ reports can account for TEs that children may have experienced before the age of explicit episodic memory consolidation and ability of retrieval. The use of the same rater for both twins likely contributed to part of the substantial proportion of variance due to the shared environment. Third, although we modeled paths of causal inference also as a framework for the next stage in our study assessing longitudinal data, these results are based on cross-sectional data. While cross-sectional data generally are not fully suitable for causal inference, twin data may reject causal hypotheses when data from unrelated persons cannot (Gillespie et al. 2003; Iacono et al. 2018; Neale et al. 1994; Verhulst and Estabrook 2012). Twin data allow modeling bi-directional causation paths, based on the cross-twin cross-trait covariances (for more details, see Duffy and Martin 1994; Heath et al. 1993; Neale et al. 1994). Model comparisons via likelihood ratio tests, using the − 2lnL and AIC estimates, consistently favored the fit and parsimony of the unidirectional model from past TEs to current PTSDsx directly and indirectly through the brain ROIs. The reverse model, where PTSDsx cause recollection of past TEs gave a much worse fit, however. When the model included additional paths (e.g., reciprocal), SEs increased and the model’s parsimony declined. The model used here represents a general framework for mediated associations between TEs and PTSDsx, which will be extended with longitudinal data. Fourth, the residualization and standardization of ordinal variables to quasi-continuous is not optimal. However, the results using normalized TEs and PTSDsx data were similar to those from our model. Furthermore, multilevel ordinal analyses may be best handled within a Bayesian modeling framework such as STAN (Carpenter et al. 2017) or its R interface Rstan (Team Stan Development 2016), but simulation and testing are required to validate its use prior to statistical genetic applications. Fifth, it is unknown if non-twin participants may have introduced consequential levels of bias in the estimation of additive-genetic and common-environmental influences in the multilevel mediation model. These issues will be addressed at later stages of this project.

## Conclusions

These findings show that if subcortical and cortical brain volumes associate indirectly with the effects of TEs on PTSDsx, they do so only to a very small or negligible extent at this age. However, we demonstrated that although the measures of TEs and PTSDsx had low endorsement rates, TEs strongly associate with PTSDsx in children 9–11 years old. Furthermore, findings from this study add to the existing literature showing that (i) an agnostic approach has the potential of uncovering brain ROIs not commonly associated with these phenotypes, and (ii) certain brain ROIs may be associated differently with TEs than with PTSDsx in children. Consistent with prior research assessing mostly adults, we found evidence that both genetic and environmental factors influence TEs, PTSDsx, and brain-imaging phenotypes in children. TEs and PTSDsx were influenced to some degree by genetic liability, but strongly influenced by common-environmental factors, and in the case of PTSDsx also by unique-environmental factors. Most of the variance in the volumes of 8 brain ROIs (from a total of 15 EN-selected brain ROIs) was accounted for by additive genetic factors. Our results may inform clinical and translational research, especially on (i) treatments focusing on environmental modifications for children at risk for developing PTSD, and (ii) mapping brain risk factors and phenotypes (e.g., structure, activation) related to trauma and PTSD phenotypes.

Our model established a framework for studying the influence of TEs directly on the development of PTSDsx, and indirectly via the volumes of brain ROIs longitudinally in children. This model and the longitudinal design extensions can be used to study other phenotypes, direction of causation (e.g., if reduced brain volumes have a causal effect on PTSDsx or vice versa), and are sufficiently powered to detect even very small effects using the large sample size of the multisite ABCD Study®. We employed an agnostic machine learning regularization approach for data reduction and variable selection among numerous brain-imaging phenotypes, integrated with multilevel (twin, siblings and unrelated individuals) structural equation modeling. This approach has the potential to identify changes in the influence of genetic and environmental sources of phenotypic variance across time, and influential associations from a large set of possible variables.

## Supplementary Information

Below is the link to the electronic supplementary material.

Supplementary file1 (PDF 1818 kb)

Supplementary file2 (PDF 345 kb)

Supplementary file3 (CSV 1 kb)

Supplementary file4 (CSV 11 kb)

Supplementary file5 (CSV 6 kb)

Supplementary file6 (CSV 40 kb)

## Data Availability

Due to privacy policies and protection of participants, ABCD Study® data will not be available here. To request access follow: https://nda.nih.gov.

## Code availability

https://github.com/dbustamantep/mlSEMhybridEN.git.
