# Supplementary material for: Associations Between Traumatic Stress, Brain Volumes and Post-traumatic Stress Disorder Symptoms in Children: Data from the ABCD Study
Source: Behav Genet. Author manuscript; Available in PMC 2022 Mar 1. (PMC8860798; doi:10.1007/s10519-021-10092-6)
Supplement: supplement [file NIHMS1776586-supplement-supplement.pdf]

Supplementary Information 1a

SupplementaryInformation1a.csv

Supplementary Information 1b

SupplementaryInformation1b.csv

Supplementary Information 1c

SupplementaryInformation1c.csv

Supplementary Information 1d

SupplementaryInformation1d.csv

Supplementary Information 2

Fig. S1: IRT Analyses, Item Characteristic Curves - TEs

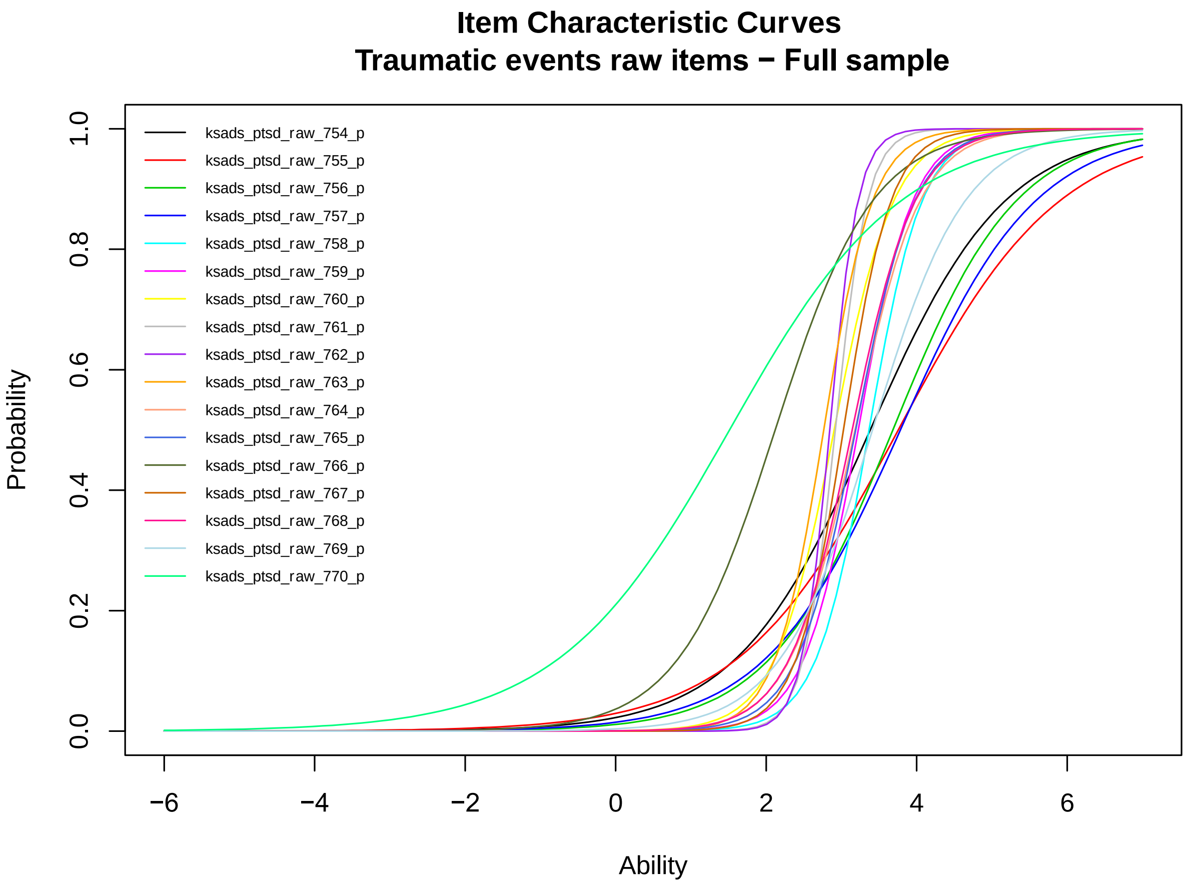

Note: IRT = Item Response Theory; TEs = Traumatic Events.

Fig. S2: IRT Analyses, Item Information Curves - TEs

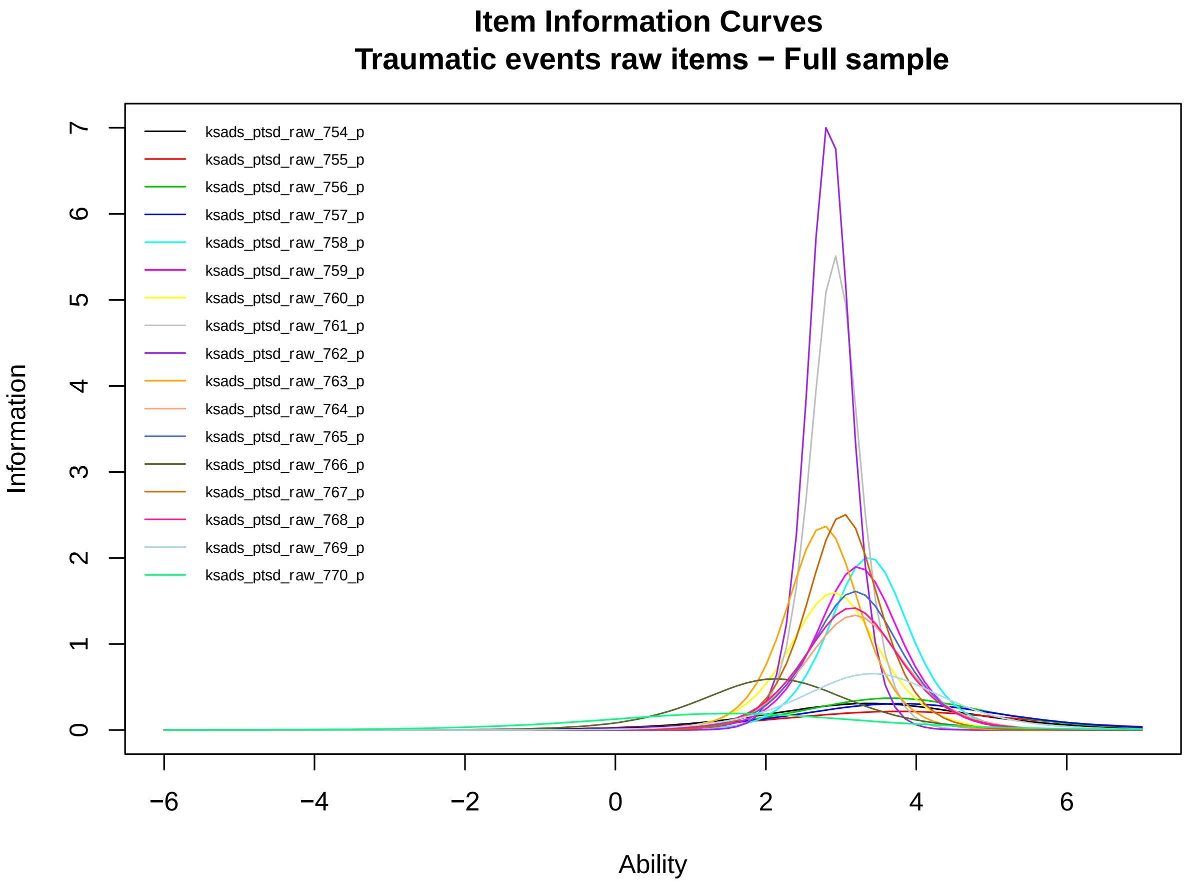

Note: IRT = Item Response Theory; TEs = Traumatic Events.

Fig. S3: IRT Analyses, Test Information and SEs - TEs

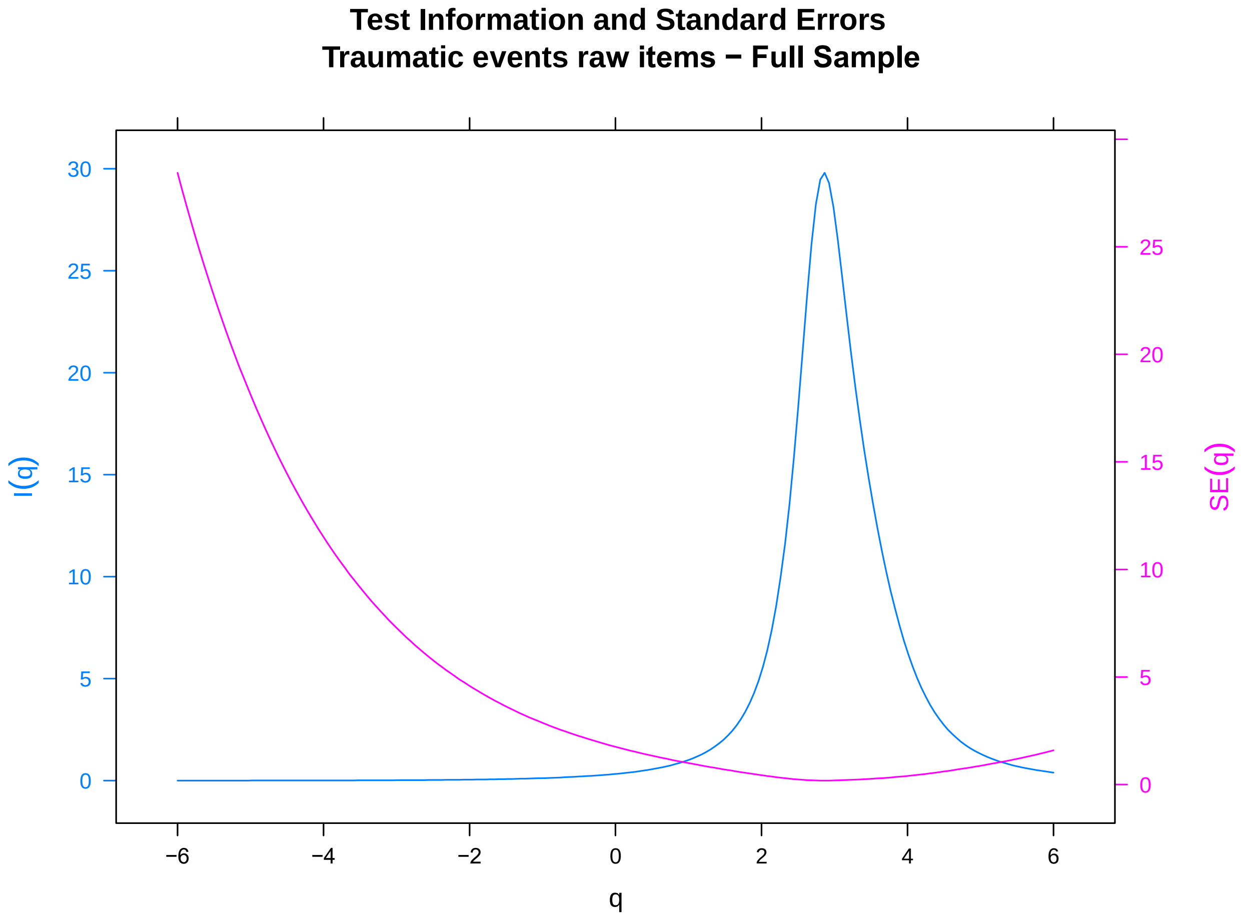

Note: IRT = Item Response Theory; SEs = Standard Errors; TEs = Traumatic Events.

Fig. S4: IRT Analyses, Item Characteristic Curves - PTSDsx

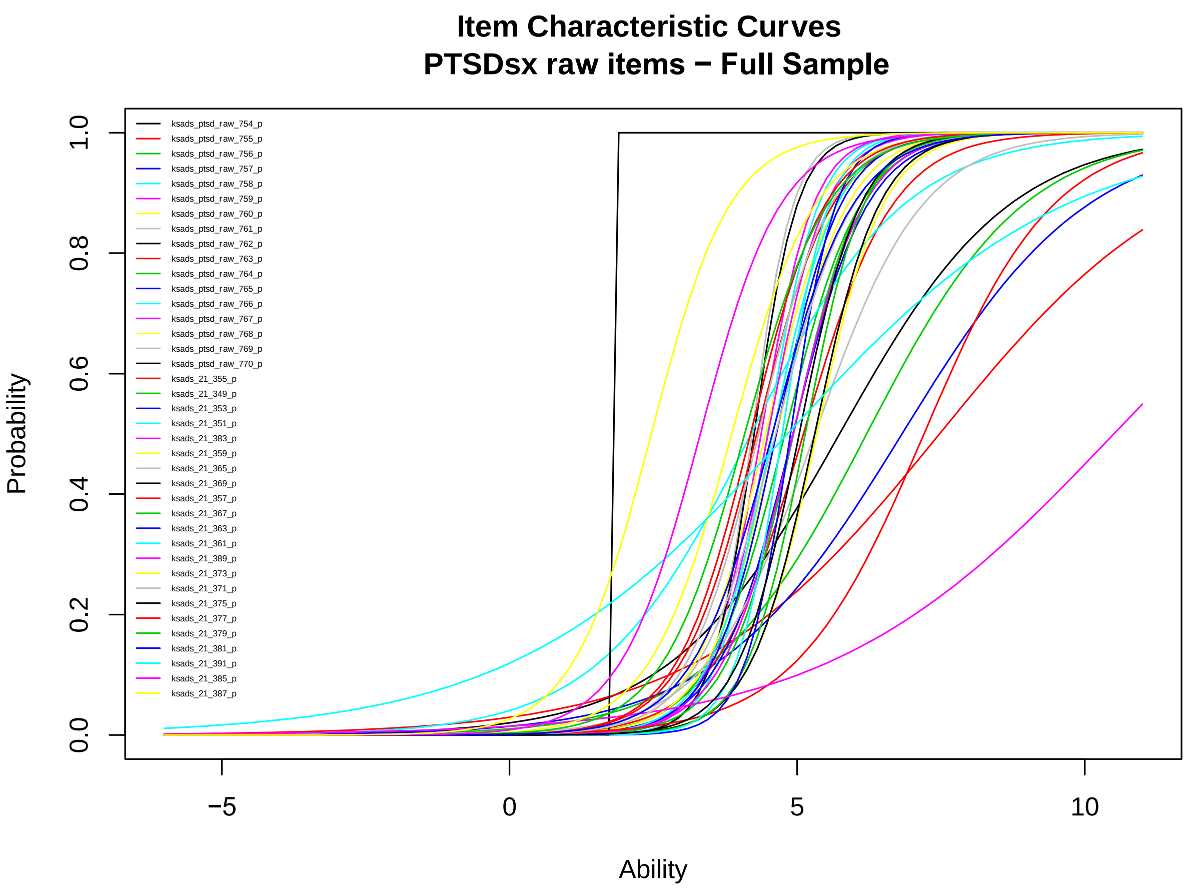

Note: IRT = Item Response Theory; PTSDsx = Post-traumatic Stress Disorder Symptoms.

Fig. S5: IRT Analyses, Item Information Curves - PTSDsx

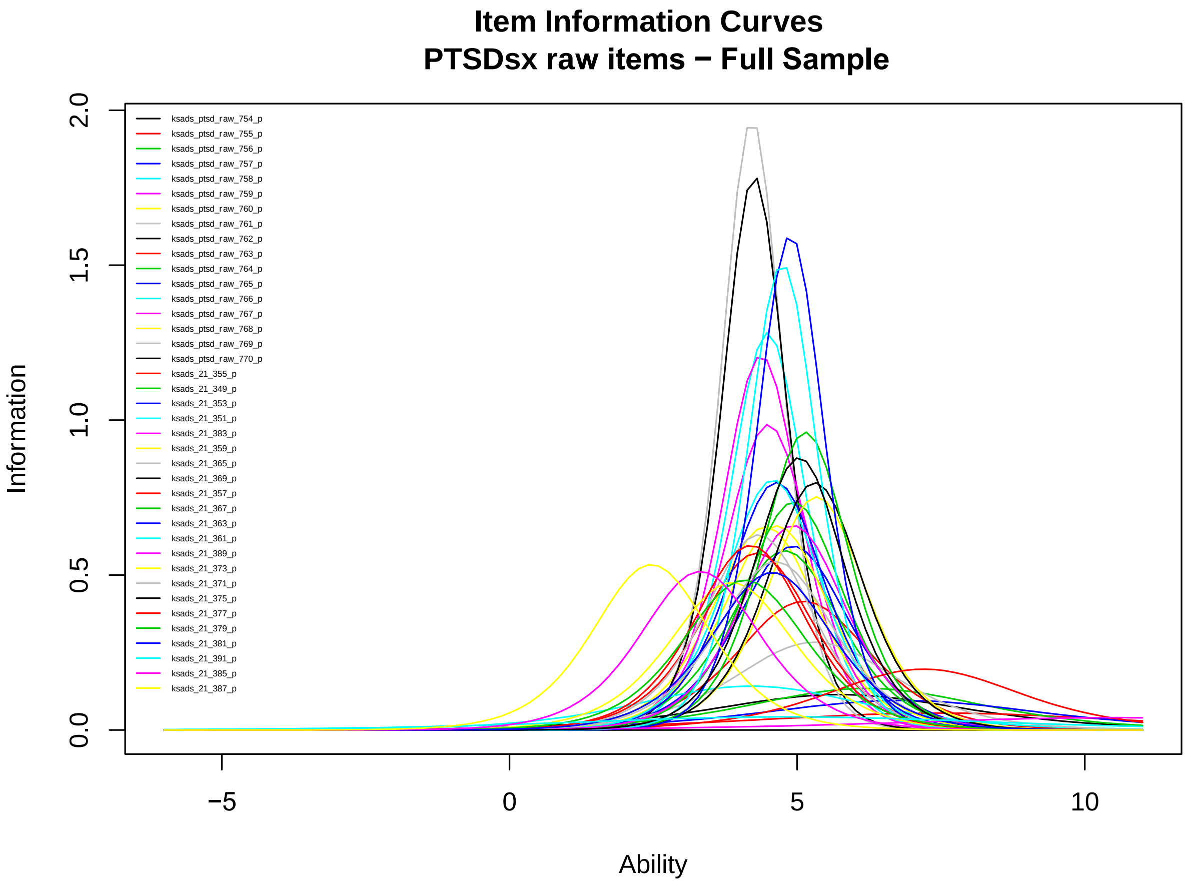

Note: IRT = Item Response Theory; PTSDsx = Post-traumatic Stress Disorder Symptoms.

Fig. S6: IRT Analyses, Test Information and SEs - PTSDsx

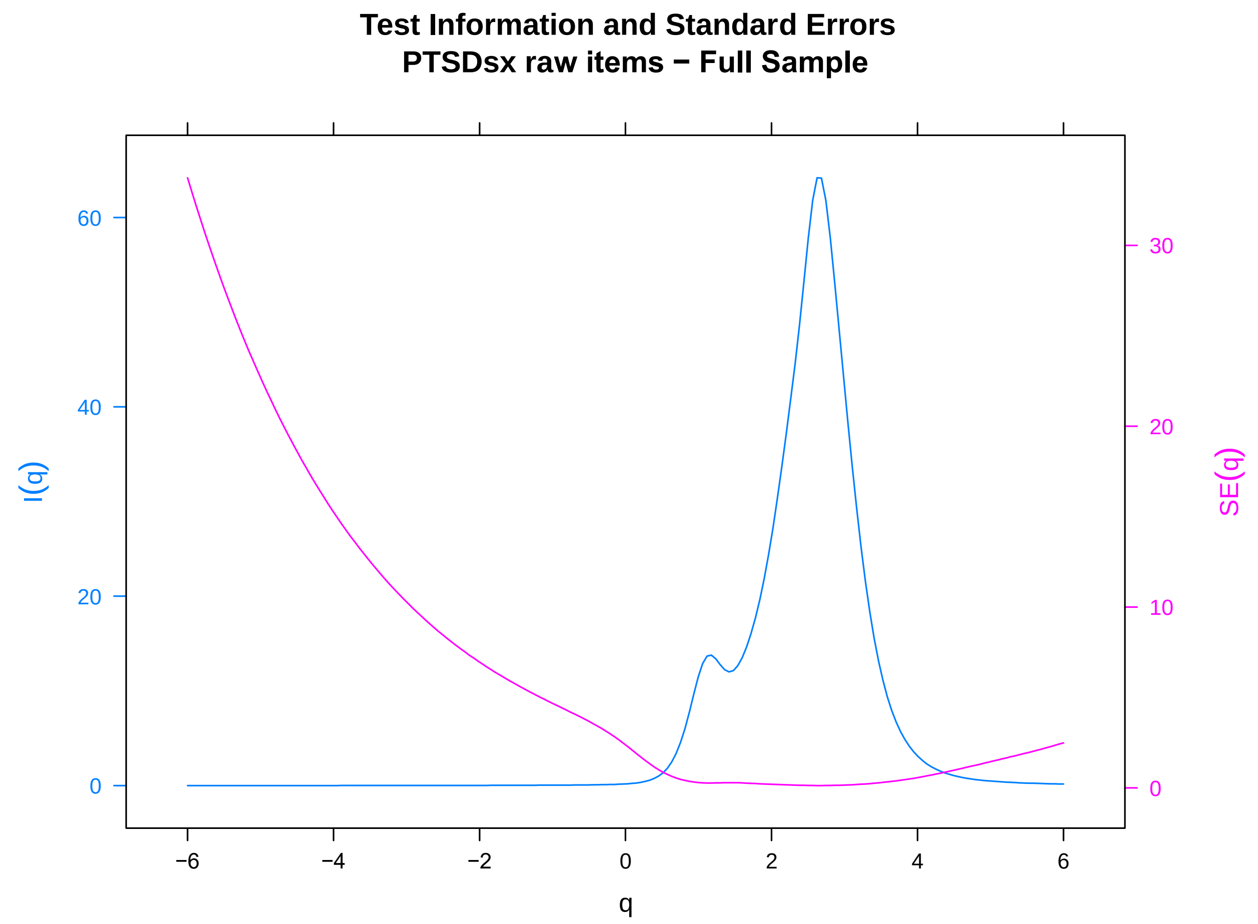

Note: IRT = Item Response Theory; SEs = Standard Errors; PTSDsx = Post-traumatic Stress Disorder Symptoms.

Fig. S7: TEs Variable pre- and post- Residualization and Standardization

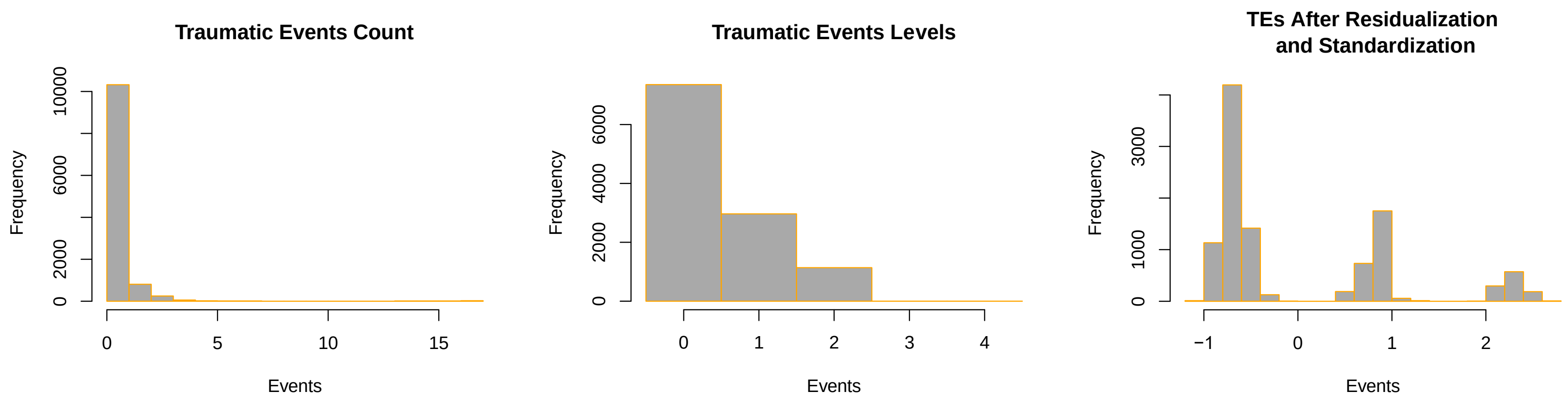

Fig. S8: PTSDsx Variable pre- and post- Residualization and Standardization

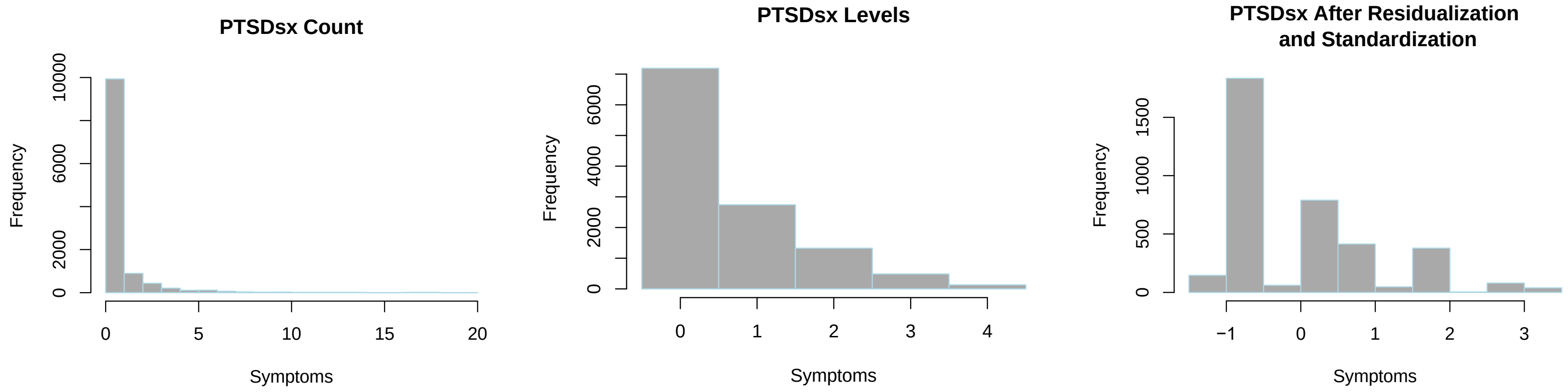

Fig. S9: Multilevel Mediation Structural Equation Model of Contralateral and Non-contralateral ROIs on Indirect Path and Reverse Paths

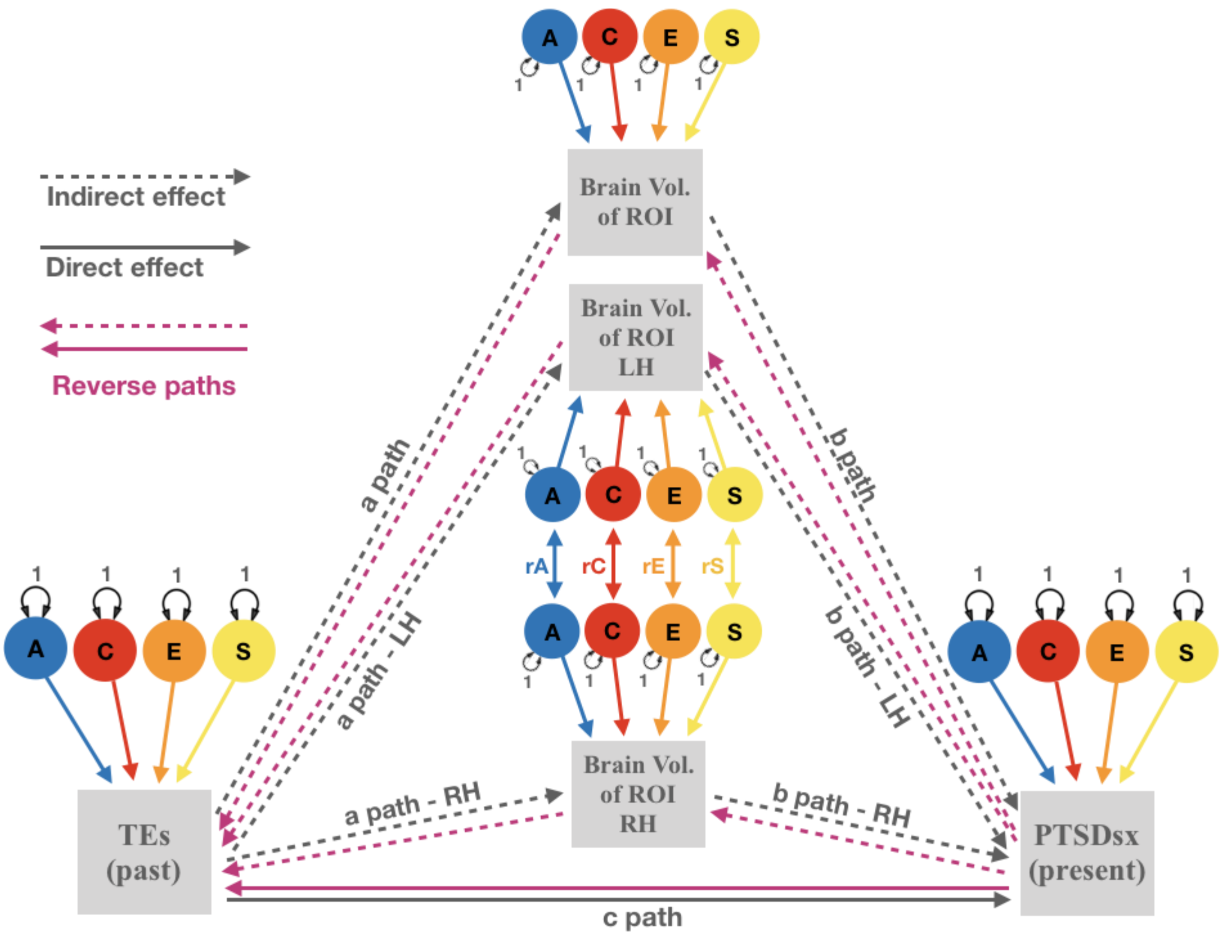

Note: A = Additive Genetic, C = Shared-Environmental, E = Unique-Environmental, S = Site factors, r = Correlation. TEs = Traumatic Events, PTSDsx = Post-Traumatic Stress Disorder Symptoms, Vol. = Volume, ROI = Region of Interest, LH = Left Hemisphere, RH = Right Hemisphere. TEs variable is modeled as the predictor, PTSDsx variable as the outcome, and brain volume of ROIs phenotypes are modeled in the indirect path (one phenotype per observed variable [denoted by a square]): i) one brain imaging phenotype (via a path and b paths), and ii) two phenotypes of the same ROI (including their variance components correlations, via a and b paths for the LH and RH).
