# Supplementary material for: Associations Between Traumatic Stress, Brain Volumes and Post-traumatic Stress Disorder Symptoms in Children: Data from the ABCD Study
Source: Behav Genet. Author manuscript; Available in PMC 2022 Mar 1. (PMC8860798; doi:10.1007/s10519-021-10092-6)
Supplement: appendix [file NIHMS1776586-supplement-appendix.pdf]

Appendix

Table A1. Variance Components Estimates (TEs, EN-selected ROIs and PTSDsx) with 95% CIs – Full and Twin Samples

	Phenotype	VA fullN	VA twinN	VC fullN	VC twinN	VE fullN	VE twinN	VS fullN	VS twinN
1	TEs	0.234 (0.19,0.27)	0.073 (-0.02,0.16)	0.616 (0.57,0.66)	0.744 (0.63,0.86)	0.151 (0.13,0.17)	0.189 (0.16,0.22)	0.007 (0.001,0.01)	0.000001 (-0.003,NA)
2	Supramarginal Gyrus, Inf. Parietal Lobe LH	0.203 (0.09,0.32)	0.335 (0.06,0.61)	0.081 (0.02,0.15)	-0.010 (-0.23,0.21)	0.713 (0.63,0.80)	0.673 (0.58,0.78)	0.004 (0.0001,0.01)	-0.001 (-0.003,0.01)
3	Subcallosal Gyrus LH	0.186 (0.06,0.31)	0.455 (0.18,0.74)	0.104 (0.04,0.17)	-0.138 (-0.36,0.07)	0.708 (0.61,0.80)	0.678 (0.59,0.79)	0.002 (-0.0005,0.01)	0.005 (-0.001,0.05)
4	Ant. Transverse Collateral Sulcus LH	0.151 (0.03,0.27)	0.350 (0.06,0.64)	0.098 (0.03,0.17)	-0.084 (-0.31,0.13)	0.748 (0.66,0.84)	0.729 (0.63,0.85)	0.003 (-0.0003,0.01)	0.004 (-0.001,0.04)
5	Inferior Temporal Sulcus LH	0.238 (0.12,0.36)	0.502 (0.23,0.79)	0.053 (-0.01,0.12)	-0.156 (-0.38,0.06)	0.705 (0.62,0.79)	0.646 (0.56,0.75)	0.003 (-0.0002,0.01)	0.004 (-0.001,0.04)
6	Anterior Cingulate Gyrus and Sulcus LH	0.290 (0.18,0.40)	0.533 (0.28,0.80)	0.136 (0.07,0.20)	0.067 (-0.28,0.14)	0.567 (0.49,0.64)	0.532 (0.46,0.67)	0.001 (0.001,0.01)	-0.002 (-0.003,0.004)
7	Medial-orbitofrontal Cortex LH	0.302 (0.18,0.42)	0.647 (0.38,0.93)	0.040 (-0.02,0.11)	-0.255 (-0.48,-0.04)	0.647 (0.56,0.73)	0.602 (0.52,0.70)	0.008 (0.002,0.02)	0.003 (-0.001,NA)
8	Occipital Lobe RH	0.528 (0.47,0.59)	0.440 (0.26,0.63)	0.339 (0.28,0.39)	0.210 (0.04,0.37)	0.133 (0.11,0.15)	0.337 (0.29,0.40)	0.007 (0.002,0.01)	0.005 (-0.001,0.05)

9	Dorsomedial Frontal Cortex RH	0.533 (0.47,0.60)	0.647 (0.43,0.88)	0.298 (0.24,0.36)	-0.043 (-0.24,0.14)	0.157 (0.13,0.18)	0.388 (0.33,0.46)	0.020 (0.01-0.03)	0.008 (-0.0001,NA)
10	Anteromedial Temporal Cortex RH	0.464 (0.40,0.53)	0.719 (0.51,0.95)	0.364 (0.31,0.42)	-0.085 (-0.28,0.09)	0.172 (0.14,0.20)	0.353 (0.30,0.41)	0.007 (0.001-0.01)	0.001 (0.001,0.08)
11	Cerebral White Matter LH	0.574 (0.51,0.63)	0.870 (0.73,1.04)	0.314 (0.26,0.37)	0.011 (-0.15,0.16)	0.114 (0.10,0.13)	0.106 (0.09,0.13)	0.004 (-0.0001,0.01)	-0.002 (-0.004,0.01)
12	Cerebellar Cortex LH	0.536 (0.48,0.59)	0.808 (NA-NA)	0.369 (0.32,0.42)	0.082 (NA,NA)	0.098 (0.08,0.11)	0.083 (NA,NA)	0.003 (-0.0003,0.01)	-0.004 (NA,NA)
13	Caudate Nucleus LH	0.544 (0.48,0.61)	0.836 (0.68,1.02)	0.292 (0.23,0.34)	0.0001 (-0.16,0.15)	0.173 (0.14,0.20)	0.16 (0.14,0.19)	0.001 (-0.001,0.003)	0.002 (-0.002,0.01)
14	Lateral Ventricle RH	0.412 (0.31,0.51)	0.867 (0.65,1.11)	0.152 (0.09,0.21)	-0.195 (-0.40,-0.01)	0.431 (0.37,0.49)	0.331 (0.28,0.39)	0.002 (-0.001,0.005)	-0.0004 (-0.003,0.02)
15	Caudate Nucleus RH	0.524 (0.45,0.59)	0.836 (0.67,1.02)	0.282 (0.23,0.33)	-0.024 (-0.19,0.13)	0.200 (0.17,0.23)	0.186 (0.16,0.22)	0.002 (-0.001,0.004)	0.0004 (-0.003,0.02)
16	Subcortical Gray Matter	0.618 (0.55,0.68)	0.829 (0.69,0.99)	0.267 (0.21,0.33)	0.049 (-0.11,0.20)	0.110 (0.09,0.13)	0.103 (0.90,0.12)	0.007 (0.002,0.01)	0.002 (-0.002,0.03)
17	PTSDsx	res:0.213 (0.09,0.32)	res:0.042 (-0.20,0.27)	res:0.184 (0.10,0.27)	res:0.267 (0.08,0.45)	res:0.218 (0.15,0.28)	res:.0.274 (0.20,0.38)	res:0.005 (-0.001,0.01)	res:-0.004 (-0.01,0.01)

Note: TEs = Traumatic Events; EN = Elastic Net; ROIs = Regions of Interest; PTSDsx = Post-traumatic Stress Disorder Symptoms; CIs = Confidence Intervals; VA = Variance Explained by Additive Genetic Factors; VC = Variance Explained by Common-environmental Factors; VE = Variance Explained by Unique-environmental Factors; VS = Variance Explained by Site Factors; LH = Left Hemisphere, RH = Right Hemisphere.

Fig. A1. Additive Genetic Correlation of TEs, PTSDsx and EN−identified Volumes of Brain ROIs

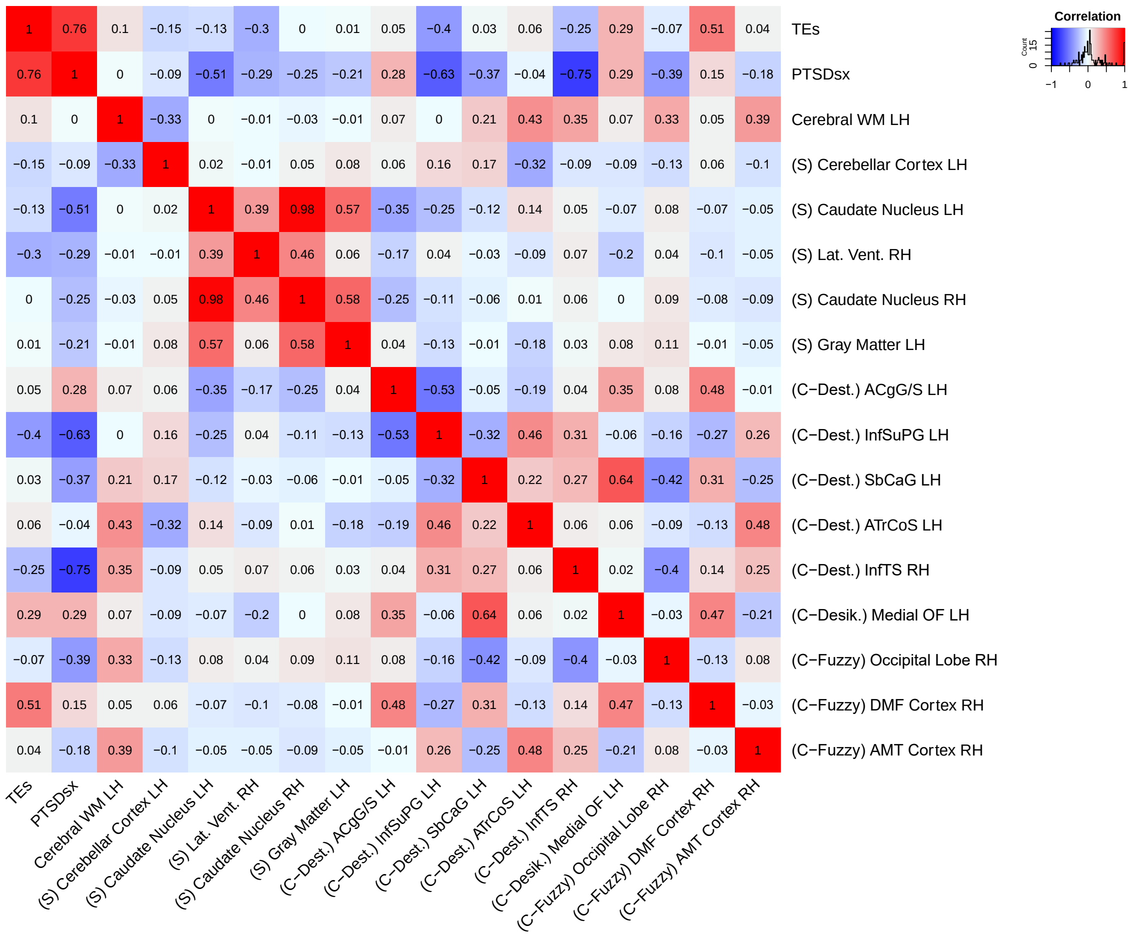

Note: TEs = Traumatic Events; PTSDsx = Post-traumatic Stress Disorder Symptoms; EN = Elastic Net; ROIs = Regions of Interest; S = Subcortical; C = Cortical; LH = Left Hemisphere, RH = Right Hemisphere; WM = White Matter; Lat. = Lateral; Vent. = Ventricle; OF = Orbitofrontal; ACgG/S = Anterior Part of the Cingulate Gyrus and Sulcus; InfSuPG = Supramarginal Gyrus of the Inferior Parietal Lobe; SbCaG = Subcallosal Gyrus; InfTS = Inferior Temporal Sulcus; AMT = Anteromedial Temporal; DMT = Dorsomedial Frontal; ATrCoS = Anterior Transverse Collateral Sulcus; Desik. = Desikan; Dest. = Destrieux.

Fig. A2. Unique-environmental Correlation of TEs, PTSDsx and EN−identified Volumes of Brain ROIs

	TEs	PTSDsx	Cerebral WM LH	(S) Cerebellar Cortex LH	(S) Caudate Nucleus LH	(S) Lat. Vent. RH	(S) Caudate Nucleus RH	(S) Gray Matter LH	(C−Dest.) ACgG/S LH	(C−Dest.) InfSuPG LH	(C−Dest.) SbCaG LH	(C−Dest.) ATrCoS LH	(C−Dest.) InfTS RH	(C−Desik.) Medial OF LH	(C−Fuzzy) Occipital Lobe RH	(C−Fuzzy) DMF Cortex RH	(C−Fuzzy) AMT Cortex RH
TEs	1	0.6	−0.03	−0.06	0.06	−0.01	−0.03	−0.05	0.01	0.03	0.04	−0.09	0	−0.06	0.07	−0.08	0.01
PTSDsx	0.6	1	−0.07	0.02	0.21	0.02	0.03	0.06	0.12	0.03	0.14	−0.06	0.11	−0.08	0.07	0.14	0
Cerebral WM LH	−0.03	−0.07	1	−0.09	−0.01	0.01	−0.03	0.18	−0.01	0.18	−0.08	0	0	−0.06	−0.04	−0.13	−0.17
(S) Cerebellar Cortex LH	−0.06	0.02	−0.09	1	−0.01	0.02	−0.04	0.01	−0.05	−0.04	−0.09	−0.02	−0.06	0.04	−0.06	−0.11	0.07
(S) Caudate Nucleus LH	0.06	0.21	−0.01	−0.01	1	0.14	0.49	0.41	−0.03	0.09	0.05	−0.02	0.01	−0.05	0.04	−0.14	0.05
(S) Lat. Vent. RH	−0.01	0.02	0.01	0.02	0.14	1	0.31	0	−0.07	−0.08	0.06	0.06	0.05	0.12	−0.03	−0.07	0
(S) Caudate Nucleus RH	−0.03	0.03	−0.03	−0.04	0.49	0.31	1	0.4	−0.11	0	0.09	−0.03	0.07	−0.02	0.03	−0.04	0.06
(S) Gray Matter LH	−0.05	0.06	0.18	0.01	0.41	0	0.4	1	−0.04	−0.01	0	−0.03	−0.01	−0.1	0.09	−0.12	0.09
(C−Dest.) ACgG/S LH	0.01	0.12	−0.01	−0.05	−0.03	−0.07	−0.11	−0.04	1	0.01	0.02	0	0.04	0.09	−0.02	0.06	0.07
(C−Dest.) InfSuPG LH	0.03	0.03	0.18	−0.04	0.09	−0.08	0	−0.01	0.01	1	−0.01	−0.08	0.07	−0.05	0.04	−0.05	−0.04
(C−Dest.) SbCaG LH	0.04	0.14	−0.08	−0.09	0.05	0.06	0.09	0	0.02	−0.01	1	−0.13	0.02	0.24	−0.02	−0.01	−0.06
(C−Dest.) ATrCoS LH	−0.09	−0.06	0	−0.02	−0.02	0.06	−0.03	−0.03	0	−0.08	−0.13	1	0.15	−0.03	−0.02	0.04	0.19
(C−Dest.) InfTS RH	0	0.11	0	−0.06	0.01	0.05	0.07	−0.01	0.04	0.07	0.02	0.15	1	0.03	0.05	−0.02	0.23
(C−Desik.) Medial OF LH	−0.06	−0.08	−0.06	0.04	−0.05	0.12	−0.02	−0.1	0.09	−0.05	0.24	−0.03	0.03	1	−0.09	0.04	−0.02
(C−Fuzzy) Occipital Lobe RH	0.07	0.07	−0.04	−0.06	0.04	−0.03	0.03	0.09	−0.02	0.04	−0.02	−0.02	0.05	−0.09	1	−0.23	0.08
(C−Fuzzy) DMF Cortex RH	−0.08	0.14	−0.13	−0.11	−0.14	−0.07	−0.04	−0.12	0.06	−0.05	−0.01	0.04	−0.02	0.04	−0.23	1	−0.08
(C−Fuzzy) AMT Cortex RH	0.01	0	−0.17	0.07	0.05	0	0.06	0.09	0.07	−0.04	−0.06	0.19	0.23	−0.02	0.08	−0.08	1

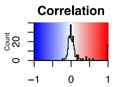

Note: TEs = Traumatic Events; PTSDsx = Post-traumatic Stress Disorder Symptoms; EN = Elastic Net; ROIs = Regions of Interest; S = Subcortical; C = Cortical; LH = Left Hemisphere, RH = Right Hemisphere; WM = White Matter; Lat. = Lateral; Vent. = Ventricle; OF = Orbitofrontal; ACgG/S = Anterior Part of the Cingulate Gyrus and Sulcus; InfSuPG = Supramarginal Gyrus of the Inferior Parietal Lobe; SbCaG = Subcallosal Gyrus; InfTS = Inferior Temporal Sulcus; AMT = Anteromedial Temporal; DMT = Dorsomedial Frontal; ATrCoS = Anterior Transverse Collateral Sulcus; Desik. = Desikan; Dest. = Destrieux.

Fig. A3. Phenotypic Correlation of TEs, PTSDsx and EN−identified Volumes of Brain ROIs

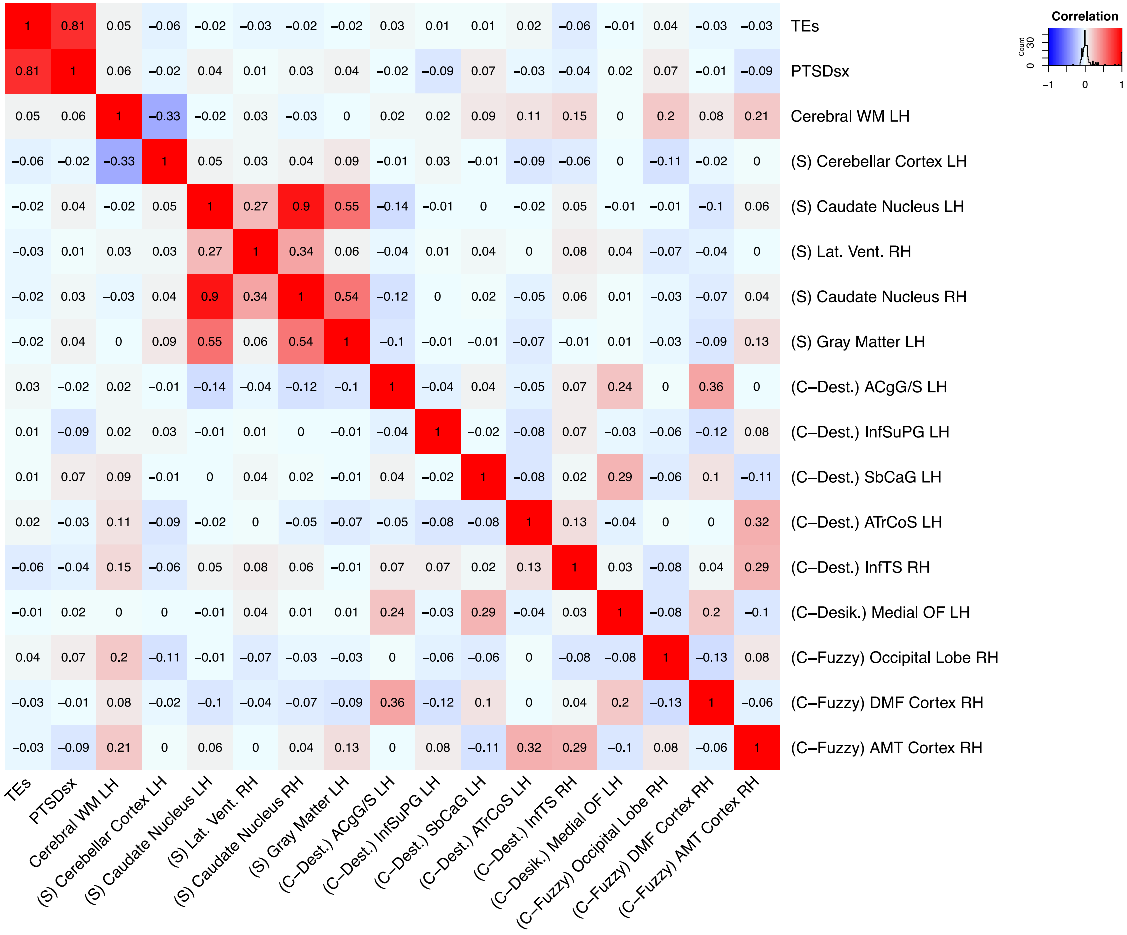

Note: TEs = Traumatic Events; PTSDsx = Post-traumatic Stress Disorder Symptoms; EN = Elastic Net; ROIs = Regions of Interest; S = Subcortical; C = Cortical; LH = Left Hemisphere, RH = Right Hemisphere; WM = White Matter; Lat. = Lateral; Vent. = Ventricle; OF = Orbitofrontal; ACgG/S = Anterior Part of the Cingulate Gyrus and Sulcus; InfSuPG = Supramarginal Gyrus of the Inferior Parietal Lobe; SbCaG = Subcallosal Gyrus; InfTS = Inferior Temporal Sulcus; AMT = Anteromedial Temporal; DMT = Dorsomedial Frontal; ATrCoS = Anterior Transverse Collateral Sulcus; Desik. = Desikan; Dest. = Destrieux.

Fig. A4. Additive Genetic Correlation of TEs, PTSDsx and EN−identified Volumes of Brain ROIs - Clustered

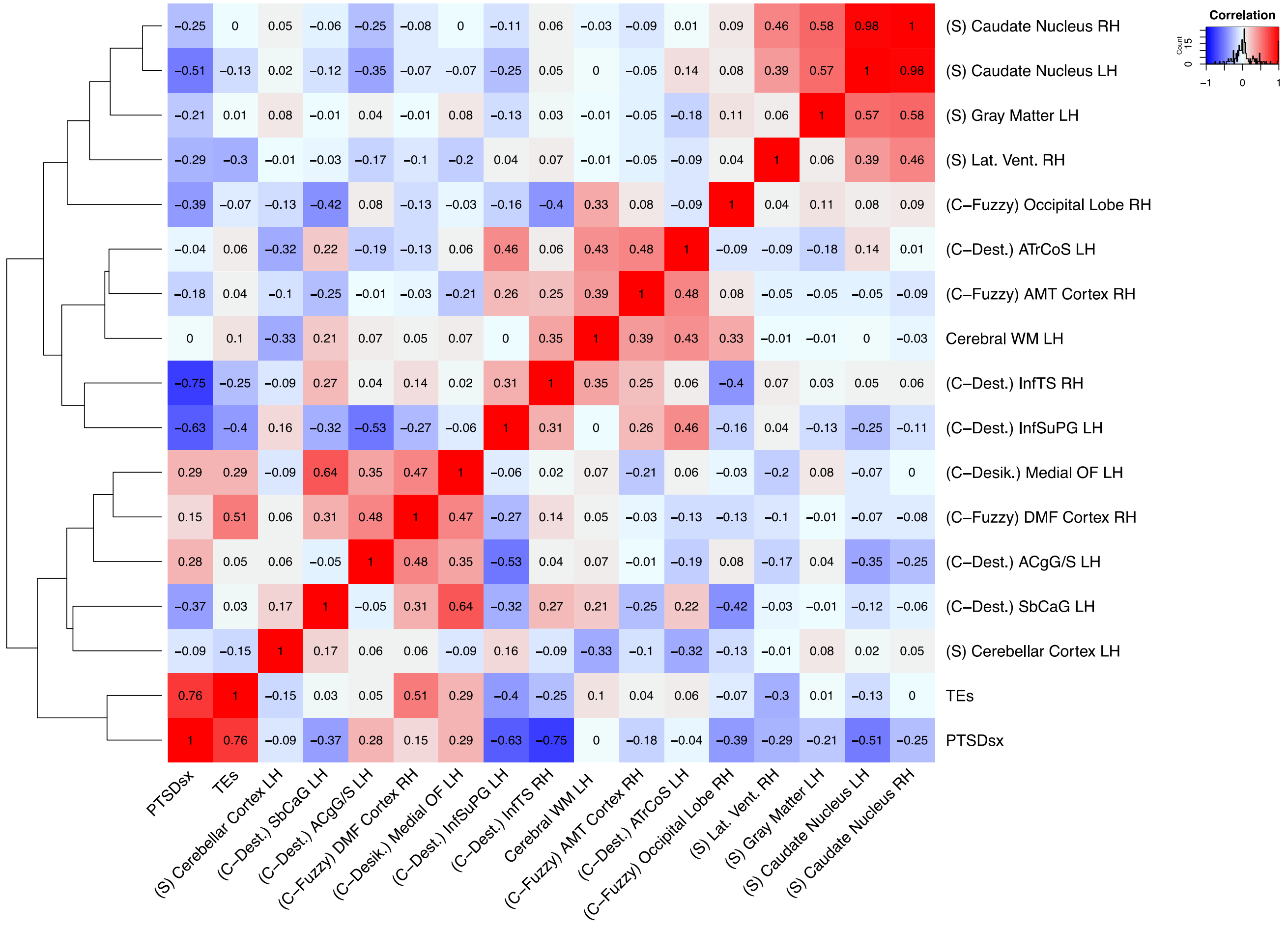

Note: TEs = Traumatic Events; PTSDsx = Post-traumatic Stress Disorder Symptoms; EN = Elastic Net; ROIs = Regions of Interest; S = Subcortical; C = Cortical; LH = Left Hemisphere, RH = Right Hemisphere; WM = White Matter; Lat. = Lateral; Vent. = Ventricle; OF = Orbitofrontal; ACgG/S = Anterior Part of the Cingulate Gyrus and Sulcus; InfSuPG = Supramarginal Gyrus of the Inferior Parietal Lobe; SbCaG = Subcallosal Gyrus; InfTS = Inferior Temporal Sulcus; AMT = Anteromedial Temporal; DMT = Dorsomedial Frontal; ATrCoS = Anterior Transverse Collateral Sulcus; Desik. = Desikan; Dest. = Destrieux.

Fig. A5. Unique-environmental Correlation of TEs, PTSDsx and EN−identified Volumes of Brain ROIs - Clustered

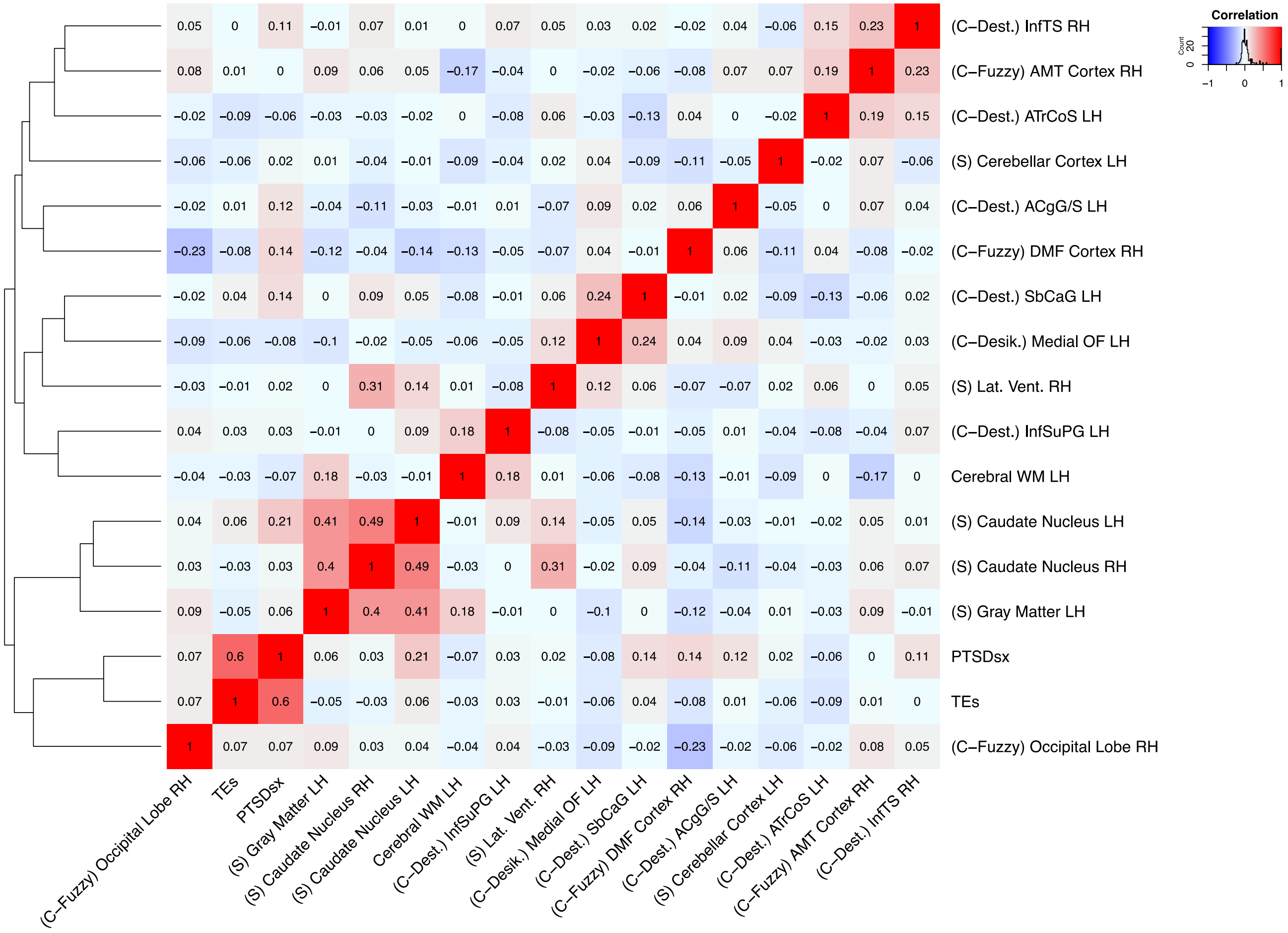

Note: TEs = Traumatic Events; PTSDsx = Post-traumatic Stress Disorder Symptoms; EN = Elastic Net; ROIs = Regions of Interest; S = Subcortical; C = Cortical; LH = Left Hemisphere, RH = Right Hemisphere; WM = White Matter; Lat. = Lateral; Vent. = Ventricle; OF = Orbitofrontal; ACgG/S = Anterior Part of the Cingulate Gyrus and Sulcus; InfSuPG = Supramarginal Gyrus of the Inferior Parietal Lobe; SbCaG = Subcallosal Gyrus; InfTS = Inferior Temporal Sulcus; AMT = Anteromedial Temporal; DMT = Dorsomedial Frontal; ATrCoS = Anterior Transverse Collateral Sulcus; Desik. = Desikan; Dest. = Destrieux.

Fig. A6. Phenotypic Correlation of TEs, PTSDsx and EN−identified Volumes of Brain ROIs - Clustered

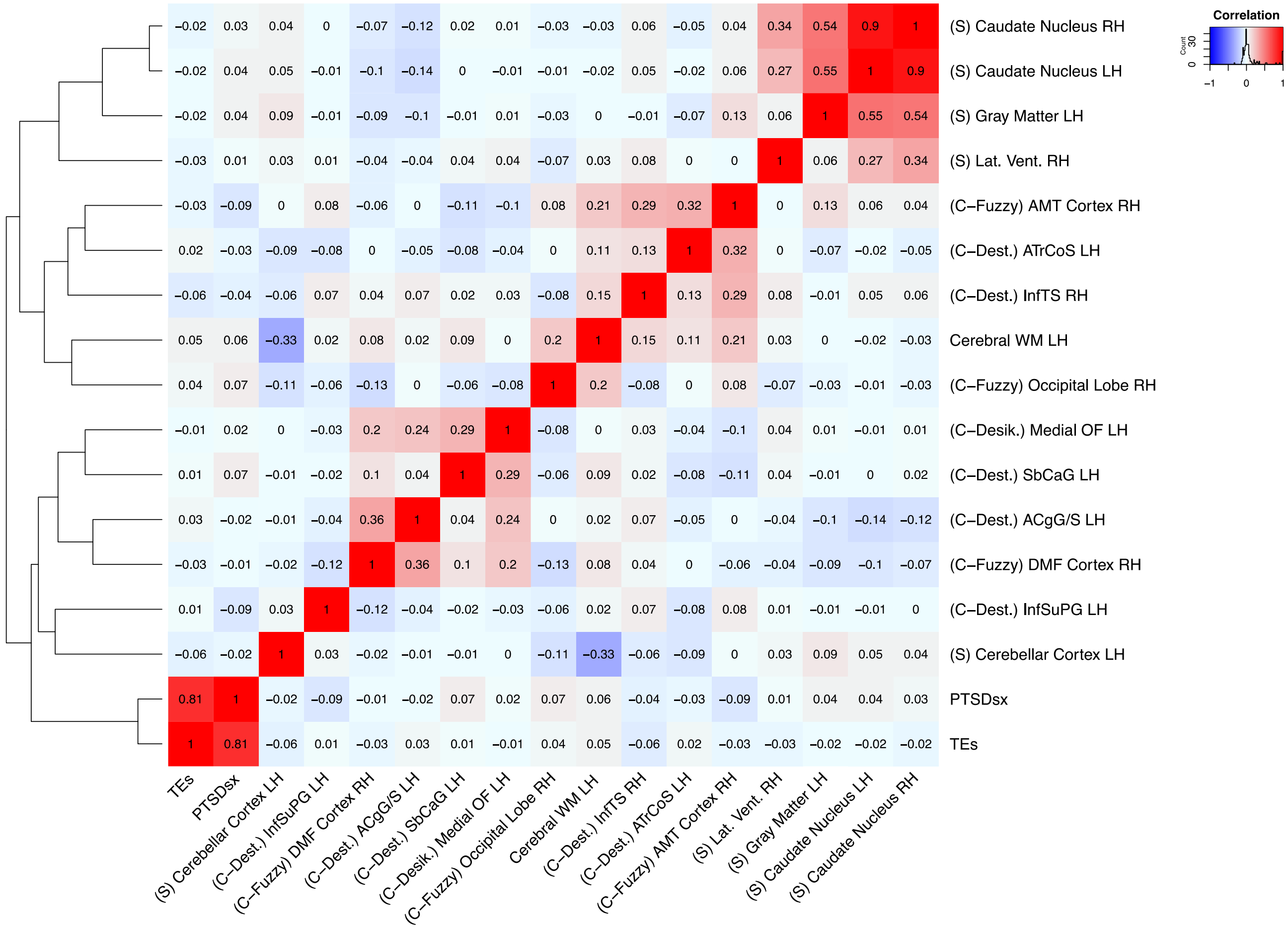

Note: TEs = Traumatic Events; PTSDsx = Post-traumatic Stress Disorder Symptoms; EN = Elastic Net; ROIs = Regions of Interest; S = Subcortical; C = Cortical; LH = Left Hemisphere, RH = Right Hemisphere; WM = White Matter; Lat. = Lateral; Vent. = Ventricle; OF = Orbitofrontal; ACgG/S = Anterior Part of the Cingulate Gyrus and Sulcus; InfSuPG = Supramarginal Gyrus of the Inferior Parietal Lobe; SbCaG = Subcallosal Gyrus; InfTS = Inferior Temporal Sulcus; AMT = Anteromedial Temporal; DMT = Dorsomedial Frontal; ATrCoS = Anterior Transverse Collateral Sulcus; Desik. = Desikan; Dest. = Destrieux.
